# The impact of enhancing nutrition and antenatal infection treatment on birth outcomes in Amhara, Ethiopia: a pragmatic factorial, cluster-randomised clinical effectiveness study

**DOI:** 10.1136/bmjgh-2024-016264

**Published:** 2025-06-18

**Authors:** Anne CC Lee, Firehiwot Workneh, Yunhee Kang, Kalkidan Yibeltal, Nebiyou Fasil, Sitota Tsegaye, Estifanos Baye, Workagegnehu Tarekegn Kidane, Yoseph Yemane Berhane, Mulatu Melese Derebe, Fred Van Dyk, Michelle Eglovitch, Ingrid Olson, Mandefro M Mengistie, Fisseha Shiferie, Tigest Shifraw, Chunling Lu, Krysten North, Grace J Chan, Sheila Isanaka, Rose L Molina, Amare Worku Tadesse, Blair J Wylie, Parul Christian, Luke C Mullany, Alemayehu Worku, Yemane Berhane

**Affiliations:** 1 Department of Pediatrics, Brown University, Providence, Rhode Island, USA; 2 Department of Pediatrics, Brigham and Women’s Hospital, Boston, Massachusetts, USA; 3 Department of Epidemiology and Biostatistics, Addis Continental Institute of Public Health, Addis Ababa, Ethiopia; 4 Center for Human Nutrition, Department of International Health, Johns Hopkins Bloomberg School of Public Health, Baltimore, Maryland, USA; 5 Department of Reproductive Health and Population, Addis Continental Institute of Public Health, Addis Ababa, Ethiopia; 6 Department of Global Health and Health Policy, Addis Continental Institute of Public Health, Addis Ababa, Ethiopia; 7 Department of Nutrition and Behavioral Science, Addis Continental Institute of Public Health, Addis Ababa, Ethiopia; 8 Amhara Public Health Institute, Bahir Dar, Ethiopia; 9 Harvard Medical School, Boston, Massachusetts, USA; 10 Division of Global Health Equity, Brigham and Women's Hospital, Boston, MA, USA; 11 Department of Pediatrics, University of Pennsylvania, Philadelphia, PA, USA; 12 Children’s Hospital of Philadelphia, Philadelphia, PA, USA; 13 Departments of Nutrition and Global Health and Population, Harvard University T H Chan School of Public Health, Boston, Massachusetts, USA; 14 Department of Obstetrics and Gynecology, Beth Israel Deaconess Medical Center, Boston, Massachusetts, USA; 15 Department of Infectious Disease Epidemiology and International Health, London School of Hygiene & Tropical Medicine, London, UK

**Keywords:** Global Health, Maternal health, Nutrition, Prevention strategies, Child health

## Abstract

**Introduction:**

We aimed to determine the impact of antenatal interventions to optimise maternal nutrition and infection management on birth outcomes in Ethiopia.

**Methods:**

We conducted a pragmatic, open-label, 2×2 factorial randomised clinical effectiveness study among pregnant women enrolled <24 weeks gestation in 12 rural health centres in Amhara, Ethiopia. Eligible health centres were randomised to deliver an enhanced nutrition package (ENP) (iron-folic acid, iodised salt and targeted micronutrient fortified balanced energy protein (BEP) supplementation for undernourished women) or routine nutrition care (iron-folic acid only). Individual women were randomised to receive an enhanced infection management package (EIMP) (genitourinary tract infection screening-treatment and enhanced deworming) or routine infection care (syndromic management). The primary outcomes were birth weight and length; secondary outcomes were gestational age, preterm delivery, small-for-gestational-age, low birth weight, stillbirth, newborn weight-for-age and length-for-age z-scores, newborn head circumference, and maternal anemia. Analysis was intention to treat.

**Results:**

From August 2020 to December 2021, 2392 women were randomised (604 ENP+EIMP, 600 ENP alone, 593 EIMP alone and 595 neither package) and followed until June 2022, with 2170 pregnancy outcomes analysed (565 ENP+EIMP, 549 ENP, 525 EIMP, 531 neither). In the ENP arm, 427 (36%) women were eligible for BEP and consumed on average 74 days. The prevalence of genitourinary tract infection was low (4.9%), while parasitic stool infections were common (31%). There was no difference in birth weight (ENP vs not-ENP: adjusted mean difference −4 g (−83 to 75); EIMP vs not-EIMP: 18 g (−35 to 70); ENP+EIMP vs neither: 14 g (−81 to 109)) or birth length (ENP: −0.3 cm (−1.1 to 0.5); EIMP: 0.2 cm (−0.1 to 0.5); ENP+EIMP: −0.1 cm (−1.2 to 1.1)) between study arms. In the ENP+EIMP group, the stillbirth rate was lower compared with the arm receiving neither package (7.1/1000 vs 24.7/1000 births; adjusted relative risk: 0.29 (0.09 to 0.94)). The packages did not significantly affect other secondary outcomes.

**Conclusions:**

In this pragmatic study implemented within the Ethiopian health system, enhanced nutrition and infection packages did not affect birth weight or length. While stillbirth rates were lower in the group receiving both packages, these findings need to be supported by additional studies.

**Trial registration number:**

ISRCTN15116516.

**WHAT IS ALREADY KNOWN ON THIS TOPIC**

- Undernutrition and infections in pregnancy are major risk factors for preterm birth and low birth weight (LBW); however, evidence on the effectiveness of interventions to prevent these vulnerable newborn types has been mixed.
- In the 2015 Cochrane systematic review, balanced energy protein (BEP) supplementation during pregnancy increased mean birth weight 41 g (11 trials; 95% CI 5 to 77 g), with greater effects in undernourished pregnant women (8 trials; 67 g, 95% CI 12 to 121 g).
- In a 2023 systematic review of antenatal interventions to reduce risk of LBW related to maternal infections in pregnancy, 15 interventions were reviewed, and screening and treatment of asymptomatic bacteriuria was deemed to potentially reduce risk of LBW (low quality of evidence).

**WHAT THIS STUDY ADDS**

- This study showed that integrated packages of WHO recommended antenatal nutrition interventions, including BEP for undernourished women, and infection management interventions implemented within the Ethiopian health system did not affect newborn birth weight or length.
- The combined delivery of both antenatal infection and nutrition intervention packages reduced the risk of stillbirth, compared with the routine care arm.
- The prenatal intervention packages increased coverage of antenatal care (ANC), with higher overall ANC contacts and coverage of at least four ANC visits in intervention arms.

**HOW THIS STUDY MIGHT AFFECT RESEARCH, PRACTICE OR POLICY**

- The multidomain, integrated approach to antenatal nutrition and infection may have reduced stillbirth risk and is an area of future research.
- Implementation research is needed to address barriers to achieve effective antenatal intervention coverage in different health systems and contexts.
- Intensified efforts to develop novel interventions and approaches to prevent small vulnerable birth outcomes are needed.

## Background

 The primary prevention of preterm birth and fetal growth restriction is one of the foremost public health challenges. In 2020, an estimated 11.9 million infants were born preterm and 23.4 million small-for-gestational-age (SGA), with ~90% of these small vulnerable newborns (SVNs, ie, preterm and/or SGA) in low-income and middle-income countries (LMIC).^1^ SVNs have a higher risk of neonatal mortality,^2^ contribute to half of neonatal deaths^1^ and carry a higher risk of impaired growth, neurodevelopment and adult chronic disease.^2^,^6^ SVNs comprise 99.5% of low birth weight infants (LBW, <2500 g), and the WHO Third Global Nutrition Target aims to reduce the proportion of infants born LBW by 30% by the year 2025.^7^ However, rates of preterm birth and LBW have remained static over the past decade.^1 8^ The 2023 Lancet SVN group has advocated for the prevention of preterm birth and fetal growth restriction as a key public health strategy to improve child survival and health.^9^ The SVN group estimated that 5.2 million SVN births could be prevented annually with the scale-up of several evidence-based interventions, including multiple micronutrient supplementation (MMS), balanced protein energy supplementation, low-dose aspirin, treatment of asymptomatic bacteriuria and syphilis, malaria prevention, smoking cessation and progesterone.^9^ However, there is a dearth of evidence from programmes that have demonstrated a beneficial impact on birth outcomes when implemented within real-world health systems.

Maternal undernutrition and infections are major, prevalent risk factors for preterm birth and fetal growth restriction in LMICs and contribute to a large population-attributable fraction of these SVN types.^10 11^ The combined exposure to undernutrition and infections in pregnancy may have synergistic adverse effects on fetal growth, development and gestational duration.^12^ Maternal undernutrition is prevalent in Ethiopia, where 23% of reproductive-age women are underweight,^13^ which is associated with increased risk of spontaneous preterm birth and LBW.^14^ Inadequate gestational weight gain during pregnancy is associated with higher risk of low birthweight and SGA.^15^ An estimated 69% of women of reproductive age are estimated to have a micronutrient deficiency,^16 17^ and trials of antenatal MMS provide causal evidence linking micronutrient deficiencies and adverse birth outcomes.^18^ Currently, antenatal MMS is recommended only in the context of rigorous research by the WHO, and in Ethiopia, iron-folic acid (IFA) continues to be standard of care. Infections are prevalent, under-recognised risk factors for adverse birth outcomes, particularly in LMICs, where routine screening and treatment of genitourinary tract infections during antenatal care (ANC) may not be performed due to resource constraints. Urinary tract infection (UTI) carries a twofold higher risk of preterm delivery.^19^ Helminthic infections increase the risk of systemic inflammation, LBW and preterm birth.^20^,^22^ Reproductive tract infections may ascend and seed the chorioamniotic membranes and amniotic fluid, predisposing to inflammation and preterm birth.^23^

Given the desire to rapidly accelerate efforts to prevent SVN, there is a need to test comprehensive approaches targeting multiple domains and maternal risk factors influencing fetal growth and gestational duration. The WHO released ANC recommendations in 2016 on an evidence-based, core package of interventions to optimise the pregnancy experience and outcomes.^24^ These recommendations include IFA, context-specific supplementation with balanced energy protein (BEP) in undernourished settings, and screening and treatment of certain pregnancy infections. In Ethiopia, not all WHO recommendations have been adopted or achieved high coverage in routine ANC, and there is a need for pragmatic studies to examine the impact of implementing these intervention packages within real-world health systems. Given the roles and interactions of nutrition and infections in pregnancy, we hypothesised that holistic and comprehensive ANC packages targeting both nutrition and infection management would result in greater benefits on pregnancy outcomes.

## Objective

We aimed to determine the impact of antenatal intervention packages to optimise maternal nutrition and/or treat maternal pregnancy infections delivered through existing routine ANC on birth outcomes in Amhara, Ethiopia.

## Methods

The Enhancing Nutrition and Antenatal Infection Treatment (ENAT) study was prospectively registered at ICRCTN (ISRCTN15116516) and led by an investigative team at Addis Continental Institute of Public Health (ACIPH), Harvard Medical School and Johns Hopkins Bloomberg School of Public Health.^25^ The protocol has been previously detailed elsewhere.^7^

### Study design

**Figure 1:**
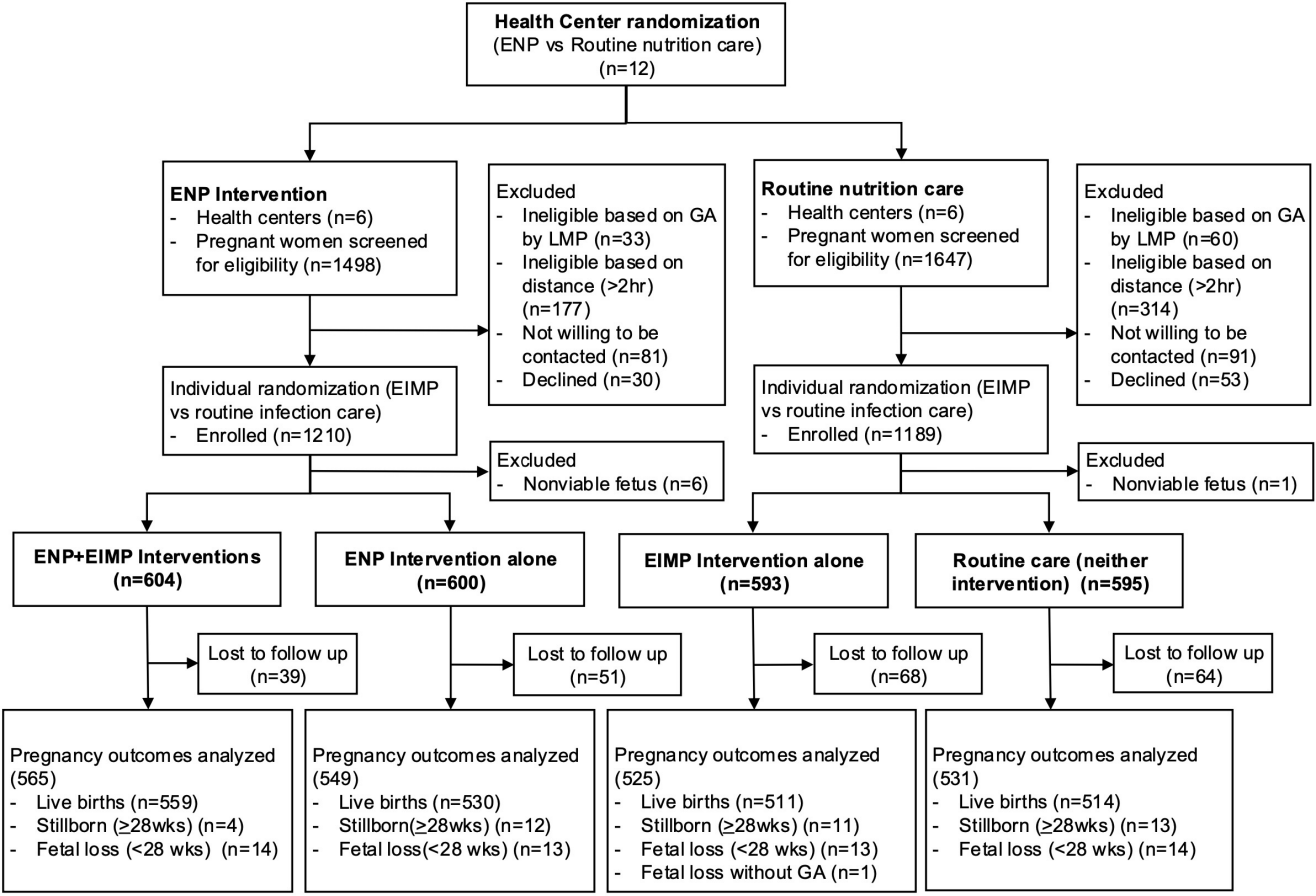
CONSORT diagram of participants in the ENAT Study. CONSORT, Consolidated Standards of Reporting Trials; ENAT, Enhancing Nutrition and Antenatal infection Treatment; EIMP, enhanced infection management package; ENP, enhanced nutrition package; GA, gestational age; LMP, last menstrual period.

The study was a 2×2 factorial pragmatic, open-label, randomised clinical effectiveness study with cluster randomisation of the enhanced nutrition package (ENP) vs routine nutrition care, and individual level randomisation of an enhanced infection management package (EIMP) vs routine infection care (figure 1). Thus, individual participants were effectively randomised to one of four study arms: ENP+EIMP intervention, ENP intervention only, EIMP intervention only or neither intervention (routine care). A cluster design was required in the local context to reduce nutrition intervention contamination in the small, rural communities and because it was not considered ethical to randomise some women within the same catchment area to receive a nutritional supplement while others did not.

### Patient and public involvement

Prior to the study, formative research (in-depth interviews) was conducted with a range of community members (mothers, families, community and religious leaders, health providers).^26 27^ This feedback directly informed the design of the study interventions, packages and their implementation. Community sensitisation was also performed prior to initiating the study.

### Study participants and recruitment

The study was conducted in 12 health centres (each serving ~25 000 population) in West Gojjam and South Gondar, in rural northwestern Amhara (~2–6 hours from the capital, Bahir Dar). Health centres were eligible that had ANC volume >250 women/year, a functioning laboratory and were accessible from the main road and within 6 hours of drive to Amhara Public Health Institute (APHI).^7^ Health centre enrolment was done by study coordinators at ACIPH.

Pregnant women recruited from ANC clinics at study health centres were eligible if ≤24 weeks gestation. Consent and enrolment were performed by study nurses before randomisation. Women were excluded if they planned to move out of the study area before delivery, lived >2 hours walking distance from ENAT health centres, or had a non-viable fetus on enrolment ultrasound. Study enrolment began in August 2020 and continued until December 2021, with neonatal follow-up ending in June 2022.

### Randomisation and masking

Randomisation was performed at two levels. At the first level, 12 health centres (clusters) were randomised in a 1:1 allocation to one of two nutrition arms: (a) ENP or (b) routine nutrition care (six ENP centres and six routine nutrition care centres). All study participants enrolled at a specific health centre received the nutrition allocation to which that centre was randomised. We performed constrained randomisation to ensure balance for key indicators including population size, prestudy ANC coverage, births and distance to Bahir Dar. The study statistician (LCM) created all possible random sequences for health centre allocations, assessed sequences for prespecified restriction criteria, and then chose randomly from eligible allocation sequences (online supplemental figure S1). At the second level, pregnant women seeking care at each study health centre were individually randomised in a 1:1 allocation to be in one of two infection management arms: (a) EIMP or (b) routine infection care. Each health centre received a pregenerated randomisation list of sequential individual assignments to EIMP or standard care for participants enrolling at that health centre, within randomly permuted blocks of size 4, 8 or 12. Randomisation lists were generated by the study statistician (LCM) in R.^28^ The allocation was kept in a concealed, sequentially numbered sealed opaque envelope until study enrolment by a study nurse. Study nurses were directly involved in intervention delivery and not masked to the intervention arm. Outcomes assessors may or may not have been aware of the infection arm assignment given the non-blinded nature of the study.

### Health systems strengthening

Strengthening of ANC services was performed in all 12 health centres before the study in partnership with Amhara Regional Health Bureau and local partners. Health systems staff were trained in ANC guidelines and measurements (blood pressure, GA, birth weight and length),^29^ and health centres were supplied with ANC equipment (sphygmomanometers, HemoCue 301, ultrasound machines) and medications.

### Study interventions

The rationale for the selection of the study interventions has been previously detailed^7^ and was based on local epidemiology and the evidence base of intervention efficacy and/or WHO ANC recommendations.^24^ At the initiation of the study, in the Amhara region, an estimated 23% women of reproductive age had low body mass index (BMI) (<18.5 kg/m^2^)^13^ and geo-helminthic infections were prevalent (21%–44%).^29 30^ There were limited data on genitourinary infection prevalence. Study intervention packages were integrated into the existing health systems and delivered by health centre staff with research nurse oversight during routine ANC visits. Monthly ANC follow-up was recommended by the Ethiopian Ministry of Health (MOH) during the study period. Data collection and study measurements were conducted by research study nurses (table 1).

**Table 1:** ENAT study intervention packages

Nutrition management		
Component	Routine nutrition careEthiopian MOH guidelines(not-ENP, control arm)	ENAT Enhanced Nutrition Package(ENP**, intervention arm)**
Nutritional education/counselling	Routine counselling provided by ANC midwives	Nutritional counselling by video and study nurse. Messages tailored based on formative research, encompassing healthy eating, adequate pregnancy weight gain, dietary diversity, increasing protein and energy in diet, IFA, iodine, and common local misperceptions about dietary restriction in pregnancy.
Iron-folic acid (IFA)	60 mg iron/400 µg folic acid	Strengthened counselling regarding benefits and strategies for reducing side effects of IFA. Enhanced adherence monitoring and re-supply of IFA.
Iodised salt	MOH recommendation of iodine fortification of salt, though heterogeneous standardisation of market salt	High-quality, adequately iodised salt provided for households in airtight, resealable polyethylene containers at enrolment, and every ANC visit. Enhanced QC and counselling re: proper use and storage of iodised salt.
Micronutrient fortified balanced energy protein supplement (BEP)	For pregnant women with MUAC <23 cm, local Corn Soya Blend is recommended in food insecure areas however not available in study catchment area	For pregnant women with MUAC<23 cm, health centre-based distribution of micronutrient fortified Corn Soya Blend (Super Cereal) daily food supplement (200 g sachet, 784 kcal/day, 28 g protein; meeting IOM recommended levels for Vitamins A, D, E, B_2_, B_3_, B_6_, B_12_, C, Ca, Ph; see online supplemental table 1 for micronutrient concentrations) at every ~monthly ANC visit (35 sachets/month)

Infection management		
Component	Routine infection careEthiopian MOH guidelines(not-EIMP, control arm)	ENAT Enhanced Infection Management Package(EIMP, intervention arm)
Urinary tract infection/asymptomaticbacteriuria	Screen: Dipstick in second/third trimesterTreat: Symptomatic women treated with amoxicillin	Screening: Urine culture and antibiotic sensitivity testing at Amhara Public Health Institute.Treatment: Targeted antibiotic treatment of symptomatic and asymptomatic bacteriuria based on antibiotic resistance patterns.
Sexually transmitted/reproductive tract infections	Syndromic management per Ethiopian MOH guidelines*	Screening: ALL pregnant women provided self-collected mid-vaginal swab for gonorrhoea, chlamydia testing by nucleic acid amplification testing (Cepheid Xpert) Pregnant women with symptoms screened for trichomonas and bacterial vaginosis, point of care testing (TrichOSOM, BVBlue) Treatment: Chlamydia: Azithromycin 1 g; Gonorrhoea: Ceftriaxone 1 g; Trichomonas Metronidazole 2 g once; BV Metronidazole 500 mg bid×7 days. Partners treated for gonorrhoea*, chlamydia, trichomonas.*
Parasitic intestinal infections	Preventive deworming with mebendazole 500 mg recommended after first trimester	August 2020–May 2021: Deworming at both second and third trimester visits with mebendazole 500 mg or albendazole 400 mg.May 2021–June 2022: Deworming as per MOH guidelines with additional third trimester stool screen and treatment of persistent parasitic infection.

* National Guidelines for the Management of Sexually Transmitted Infections Using the Syndromic Approach, MoH Ethiopia (July 2015).

.ANC, antenatal care; bid, two times per day; BV, bacterial vaginosis; EIMP, enhanced infection management package; ENAT, Enhancing Nutrition and Antenatal infection Treatment; ENP, enhanced nutrition package; IOM, Institutes of Medicine; MOH, Ministry of Health; MUAC, mid-upper arm circumference; QC, quality control.

#### Enhanced nutrition package

The ENP included nutritional counselling, strengthening IFA delivery, a regular household supply of iodised salt and BEP supplementation for undernourished women (table 1). Nutrition counselling was locally contextualised based on our formative research.^26^ We strengthened delivery and counselling for IFA and provided a regular supply of high-quality, adequately iodised salt (Waff Manufacturing, 30–40 ppm potassium iodate, 600 g bottle) at each ANC contact. Women who met the criteria for undernutrition (mid-upper arm circumference (MUAC) <23 cm at any ANC visit) received a daily micronutrient-fortified BEP supplement (200 g; 28 g protein, 784 kcal/day) until delivery. The BEP formulation was selected in consultation with the Ethiopian MoH and was a locally produced corn soya flour blend (Super Cereal, Faffa Food Share Company, Addis Ababa and Ethiopia) that met Institutes of Medicine recommended levels^31^ for Vitamins A, D, E, B_2_, B_3_, B_6_, B_12_, C, calcium and phosphorus (online supplemental table S1). The corn soya flour blend was chosen given that it was a vegan product, familiar to the local population and had high acceptability and adherence in our pilot before initiation of the study.^32^ The BEP product was provided monthly at ANC visits (35 daily sachets). Women who missed follow-up were visited at home to remind them to return for ANC care and BEP distribution.

#### Enhanced infection management package

Women provided a mid-void clean catch urine sample for urine culture and a self-collected mid-vaginal swab for chlamydia/gonorrhoea testing (Xpert CT/NG assay (Cepheid, Sunnyvale, California, USA)) performed at APHI. Women who reported vaginal symptoms were tested at the health centre with point-of-care tests for Trichomonas (OSOM, Sekisui Diagnostics, Lexington, Massachusetts, USA) and bacterial vaginosis (BVBLUE, Gryphus Diagnostics, Knoxville, Tennessee, USA). Genitourinary tract infections were classified, managed and treated according to protocols consistent with MoH guidelines (online supplemental table S2, figures S2–S4). Women with identified infections were contacted and advised to return for treatment with the first antibiotic dose directly observed, and a test of cure at the following routine ANC visit. Deworming with mebendazole (500 mg) was provided twice in pregnancy (second and third trimester) consistent with WHO guidelines.^33^ At the mid-enrolment study monitoring committee (SMC) review (May 2021), UTI/sexually transmitted infection (STI) screening-treatment was discontinued given the low infection prevalence and supply chain shortages due to the COVID-19 pandemic. Additionally, given the low coverage of the two deworming doses, the SMC recommended modifying the study protocol to a first dose of mebendazole followed by third trimester stool microscopy screening and treatment (online supplemental table S3).

### Outcome measures

Outcomes were measured by trained research staff and case definitions are detailed in online supplemental table S4.

The primary study outcomes were newborn weight and length measured within 72 hours of birth.

Secondary outcomes included duration of gestation, proportion of pregnancies resulting in spontaneous preterm delivery, proportion of newborns born preterm, proportion of newborns born LBW, proportion of newborns born SGA, stillbirth rate, newborn weight-for-age z-scores, newborn length-for-age z-scores, newborn head circumference and maternal anaemia.

### Data collection and measurement

Data were collected by research staff at the health centre study visits (online supplemental table S5), and for births occurring outside of health facilities, a home visit was conducted within 72 hours of delivery. Data were collected on tablets using Survey Solutions (World Bank, V.20.08, 2021) with programmed validity checks and routine data quality control checks.

Transabdominal obstetric ultrasound (GE Vscan Access, General Electric, Boston, Massachusetts, USA) was performed by a trained research nurse for pregnancy dating. Sonographers from GE, the Ethiopian Radiography Association and Harvard Medical School performed ultrasonography training and standardisation. Crown-rump length, biparietal diameter, head circumference, femoral length, and abdominal circumference were measured in duplicate. Enrolment GA was determined by a hierarchical algorithm (online supplemental table S6). For quality control, 10% of images were externally reviewed (BJW). Adherence to nutritional supplements was assessed at ANC and birth visits by maternal recall (prior 7 days) and a physical count of returned pill bottles or consumed/empty sachets. Postnatal visits were made at 4–6 weeks for all participants to collect data on maternal and infant vital status, health, morbidity and anthropometrics.

Research staff were trained and standardised in anthropometric measurements using INTERGROWTH-21st standard operating procedures.^34^ We equipped delivery rooms with digital infant weight (ADE M112600, Germany; precision 5 g) and length scales (Perspective Enterprises PE-RILB-LTWT, Michigan USA, measures to nearest 1 mm), and trained health centres’ delivery room staff.^35^ Head and MUAC were measured to the nearest mm using insertion tapes. Daily equipment calibration checks were done before measurements.^36 37^ Haemoglobin was measured at follow-up ANC visits (second and third trimester) using HemoCue (801, Angeholm Sweden).

### Sample size

We estimated enrolling 2400 pregnant mothers across 12 health centres over 18 months, yielding 1440 infants with primary outcomes. We calculated the effect size detectable at prespecified power levels, based on previously published data regarding infant size in the region,^38^,^40^ and coefficients of variation (weight k=0.01; length k=0.008).^38^ We had 80% power to detect a 77 g birth weight and 3.0 mm length difference between the EIMP versus routine infection care arms, and a 99 g birth weight and 7.8 mm length difference between the ENP vs routine nutrition care arms.

### Statistical analysis

The statistical analysis plan was published online.^7 25^ We conducted descriptive analyses of variables at the individual and cluster levels to assess randomisation balance and intervention coverage. We followed intention-to-treat principles and used factorial analysis to determine the marginal intervention effects in the absence of interaction. Specifically, we determined the effect of the ENP package by comparing all ENP arms (ie. ENP+EIMP and ENP alone) vs routine nutrition care arms (ie. ‘not-ENP,’ EIMP and neither intervention), and the EIMP effect by comparing all EIMP arms (ie. ENP+EIMP and EIMP alone) vs routine infection care arms (ie. ‘not-EIMP,’ ENP alone and neither intervention). We also examined the combined effect of the ENP+EIMP package compared with neither intervention. A priori, we decided not to test for interaction because we considered an additive effect of the packages of public health importance and were not adequately powered to detect an interaction effect. The study had two co-primary outcomes, newborn weight and length. We report 97.5% CIs for the coprimary outcomes, reflecting a Bonferroni adjustment^41^ for multiple comparisons. Effect estimates were adjusted for prespecified baseline variables prognostic of birth size as recommended by Consolidated Standards of Reporting Trials^7^ and those that were imbalanced by study arm. We used cluster-level analysis^42^ to estimate the effect of the ENP intervention given the small number of clusters (six per group). We estimated the mean adjusted cluster birthweights and compared the distribution using a t-test. For dichotomous outcomes, we calculated covariate-adjusted cluster event rates with log-binomial regression. To estimate the marginal effects of EIMP, we used multivariate linear regression for continuous and log-binomial regression for dichotomous outcomes, with robust variance accounting for health centre clustering. To assess the effect of the combined ENP+EIMP package, we used generalised estimating equations. For newborn size metrics, z-scores were calculated using the Intergrowth Newborn size standards.^43^ Haemoglobin was adjusted for altitude (study sites: ~2000 m) per WHO guidelines^44^ and anaemia defined as <110 g/L.

We explored potential differential effects of the intervention by testing for interaction between the intervention and the following prespecified subgroups: maternal BMI (<18.5 kg/m^2^), age (<20 yo) and parity (nulliparous). Adherence to the BEP supplement was calculated as the proportion of eligible days the woman consumed BEP. Among the population with maternal MUAC<23 cm, we conducted a prespecified per-protocol analysis for women with higher adherence (>50% of eligible BEP days). In sensitivity analyses, we conducted multiple imputation to impute missing outcome measures at birth (weight and length) using the a priori predictors of birth size that were significantly associated with the outcomes (maternal age, height, BMI, infant sex and GA at birth) using previously described methods.^45^ A total of 20 imputations of missing values were done for those with missing birth anthropometrics. Estimates of effect size using imputed outcomes were estimated by following the same methods as above on each of the 20 imputed datasets and combining the individual estimates using Rubin’s rules.^46^

### Study monitoring

The SMC included an Ethiopian obstetrician and an international biostatistician with clinical trial expertise, who met before study initiation, at the enrolment midpoint, and after study completion.

#### Role of funding source

The funder provided inputs on the study design and interventions; however, did not participate in data collection, data analysis, interpretation of data, writing of the report or decision to submit the report for publication.

## Results

### Study participants

From 3 August 2020 to 10 December 2021, we screened 3145 women for eligibility and enrolled 2399 pregnant women. Excluding pregnancies with a non-viable fetus (n=7), 2392 women were randomised into 4 study arms: (1) ENP+EIMP (604), (2) ENP alone (600), (3) EIMP alone (593) or (4) neither intervention (595) (figure 1). The last postpartum visit was conducted on 4 June 2022. 2170 pregnancies were followed until delivery with 2209 infant birth outcomes analysed. There were 2114 live births, among whom 1044 were male and 1033 female (37 missing sex data). The flow diagram of participant follow-up by study arm is shown in figure 1.

Randomisation balance is shown in table 2 for the nutrition study arms (ENP vs routine nutrition care arm), table 3 for infection study arms (EIMP vs routine infection care arm) and online supplemental table S7 for the cluster (health centre) level. Nutrition arms were imbalanced on several maternal baseline characteristics, as defined by >3% difference.^47^ The ENP arm was more disadvantaged than the routine nutrition care arm at baseline, with a lower prevalence of land ownership and educational attainment, and a higher prevalence of agricultural labour and maternal undernutrition (maternal BMI <18.5 kg/m^2^). Participant characteristics were balanced across infection arm group allocation (table 3).

**Table 2:** Pregnancy level baseline randomisation balance by nutrition arm allocation

	**InterventionENP** (N=1204)	**Controlnot-ENP** (Routine nutrition care) (N=1188)
Age, mean (SD), years	26.1 (5.6)	26.5 (5.5)
Maternal parity		
0	361 (30.1)	351 (29.7)
1	840 (69.9)	833 (70.4)
Missing data—no	3	4
Education, n (%)		
No education	590 (49.5)	563 (47.1)
Primary	357 (30.0)	314 (26.6)
Secondary or higher	244 (20.5)	303 (25.7)
Missing data—no.	13	8
Marital status (married), n (%)	1141 (96.2)	1134 (95.6)
Missing data—no.	18	2
Occupation, n (%)		
No formal occupation	292 (24.5)	378 (32.0)
Agriculture/daily labour	673 (56.5)	531 (45.0)
Wage occupation (merchant, government)	227 (19.0)	271 (23.0)
Missing data—no	12	8
Land ownership, n (%)	812 (68.2)	848 (71.9)
Missing data—no	13	9
Animal ownership, n (%)	763 (64.1)	729 (61.8)
Missing data—no.	13	9
Fuel access/use, n (%)	18 (1.5)	37 (3.1)
Missing data—no.	14	9
Toilet/water access, n (%)	975 (81.9)	930 (78.9)
Missing data—no	14	9
Food insecurity, n (%)		
Secure	1063 (89.3)	1061 (90.0)
Mildly insecure	57 (4.8)	69 (5.1)
Moderate-severely insecure	70 (5.9)	58 (4.9)
Missing data—no.	14	9
GA at enrolment, mean (SD), wks	16.4 (5.6)	16.2 (5.7)
Missing data—no.	2	1
Enrolment maternal MUAC<23 cm, n(%)	340 (28.5)	313 (28.9)
Missing data—no.	9	105
Enrolment maternal BMI, n (%), kg/m^2^		
<18.5	231 (19.4)	170 (14.5)
18.5–24.9	913 (76.5)	943 (80.3)
≥25	49 (4.1)	62 (5.3)
Missing data—no.	11	13
Enrolment maternal height, mean (SD), cm	157.9 (6.0)	157.8 (5.6)
Missing data—no.	11	11

BMI, body mass index; ENP, enhanced nutrition package; GA, gestational age; MUAC, mid-upper arm circumference.

**Table 3:** Pregnancy level baseline randomisation balance by infection arm allocation

	**InterventionEIMP** (N=1197)	**Controlnot-EIMP** (routine infection care)**(n=1195)**
Age, mean (SD), years	26.2 (5.7)	26.4 (5.5)
Maternal parity		
0	365 (30.6)	347 (29.1)
1	828 (69.4)	845 (70.9)
Missing data—no.	4	4
Education, n (%)		
No education	577 (48.7)	576 (48.6)
Primary	340 (28.7)	331 (27.9)
Secondary or higher	268 (22.6)	279 (23.5)
Missing data—no.	12	9
Marital status (married), n (%)	1141 (96.2)	1134 (95.6)
Missing data—no.	11	9
Occupation, n (%)		
No formal occupation	339 (28.6)	331 (27.9)
Agriculture/daily labour	604 (50.9)	600 (50.6)
Wage occupation (merchant, government)	243 (20.5)	255 (21.5)
Missing data—no.	11	9
Land ownership, n (%)	834 (70.4)	826 (69.7)
Missing data—no.	13	9
Animal ownership, n (%)	749 (63.3)	743 (62.7)
Missing data—no.	13	9
Fuel access/use, n (%)	28 (2.4)	27 (2.3)
Missing data—no.	12	11
Toilet/water access, n (%)	957 (80.8)	948 (80.1)
Missing data—no.	12	11
Food insecurity, n (%)		
Secure	1072 (90.5)	1052 (88.9)
Mildly insecure	54 (4.6)	63 (5.3)
Moderate-severely insecure	59 (5.0)	69 (5.9)
Missing data—no.	12	11
GA at enrolment, mean (SD), wks	16.2 (5.7)	16.4 (5.6)
Missing data—no.	3	0
Enrolment maternal MUAC<23 cm, n (%)	329 (28.8)	324 (28.6)
Missing data—no.	53	61
Enrolment maternal BMI, n (%), kg/m^2^		
<18.5	196 (16.6)	205 (17.3)
18.5–24.9	925 (78.2)	931 (78.6)
≥25	62 (5.2)	49 (4.1)
Missing data—no.	14	10
Enrolment maternal height, mean (SD), cm	157.8 (5.7)	157.9 (5.9)
Missing data—no.	12	10

BMI, body mass index; EIMP, enhanced infection management package; GA, gestational age; MUAC, mid-upper arm circumference.

### Intervention coverage and adherence

The ENP arm that included nutrition supplements had a higher number of ANC visits and proportion of women who received four or more antenatal contacts (ENP: 72.1%, not-ENP: 28.6%). In the ENP arm (table 4), 427 (35.7%) of women had a MUAC <23 cm on any ANC contact, and BEP was distributed an average of 2.8 times (SD 1.7) covering 3 months of pregnancy. Women reported an average of 73.7 (SD 54.8) days of BEP consumption during the pregnancy, or 52% of eligible days. BEP was not available at the routine nutrition care health centres during the study period. IFA adherence was higher (p<0.05) in the ENP arm (ENP: 74.3 days (SD 43.8), with 51.5% of women with >50% adherence; not-ENP: 62.9 days (SD 38.4), 36.1% with >50% adherence). In the ENP arm, iodised salt was distributed an average of 4.0 (SD 1.6) times.

**Table 4:** ENAT nutrition intervention delivery and compliance

	InterventionENP(n=120)	Controlnot-ENP (routine nutrition care)(n=1188)
Number of ANC Visits, mean (SD)	4.3 (1.7)	2.8 (1.2)
At least 4 ANC visits, n (%)	868 (72.1)	340 (28.6)
Balanced energy protein (BEP) supplement		
BEP delivery		
MUAC*<23 cm identified on any ANC visit, n (%)	427 (35.7)	396 (35.0)
Women distributed BEP on any ANC visit, n (%)	418 (97.9)	–
Number of BEP distributions (1 month supply), mean (SD)	2.8 (1.7)	–
BEP adherence		
Days consumed BEP†, mean (SD)	73.7 (54.8)	–
% of eligible days BEP consumed†, mean (SD)	52.0% (32.8)	–
Consumed >50% of BEP‡, n (%)	180 (52.5)	–
Consumed >75% of BEP‡, n (%)	98 (28.6)	–
Iron folic acid (IFA)		
Number IFA tablets distributed, mean (SD)	85.8 (39.2)	82.3 (41.5)
IFA adherence§		
Days consumed IFA, mean (SD)	74.3 (43.8)	62.9 (38.4)
% of eligible days IFA consumed, mean (SD)	48.6 (28.9)	41.4 (26.3)
Consumed >50% of IFA, n (%)	562 (51.5)	351 (36.1)
Consumed >75% of IFA, n (%)	211 (19.3)	108 (11.1)
Iodised salt		
Distributed iodised salt at first visit, n (%)	1193 (99.1)	–
Times iodised salt distributed, mean (SD)	4.0 (1.6)	–
Times consumed iodised salt in past 24 hours¶, mean (SD)	2.4 (1.0)	1.3 (1.4)
Nutrition counselling		
Ever-received nutrition counselling, n (%)	773 (64.2)	548 (46.1)

* MUAC measured in at least one ANC visit in n=1195 in ENP arm; n=1130 in not-ENP arm.

† BEP adherence data available until birth for 241 participants.

‡ BEP adherence data available for 343 participants until last ANC visit.

§ IFA adherence data available for 1092 in ENP arm; 973 in not-ENP arm.

¶ Average consumption for available ANC visits; iodised salt consumption data available in 1101 in ENP; 968 in not-ENP arm.

ANC, antenatal care; ENAT, Enhancing Nutrition and Antenatal infection Treatment; ENP, enhanced nutrition package; GA, gestational age; MUAC, mid-upper arm circumference.

Women in the EIMP arm had higher rates of deworming than those in the routine infection care arm (78% vs 55% with at least single dose), and during the stool screening period, 31% (109/350) had ova or parasitic infections (table 5), most commonly *Giardia lamblia* (n=43/109, 39%) and *Entamoeba histolytica* (n=40/109, 37%). The prevalence of any genitourinary tract infection overall was 4.9%. UTI prevalence was 3.5% (21/605), and STI were very rare with only 2 (0.3%) cases of chlamydia identified. The prevalence of symptomatic bacterial vaginosis and trichomonas was also very low at 0.8% and 0.2%, respectively.

**Table 5:** ENAT infection management intervention delivery and compliance

	InterventionEIMP(n=1197)n (%)	Controlnot-EIMP (routine infection care)(n=1195)n (%)
Stool parasitic infections		
Presumptive deworming		
Received one dose (mebendazole)	709 (59.2)	593 (49.5)
Received two doses (mebendazole)	225 (18.8)	69 (5.8)
Received at least single dose	934 (78.0)	662 (55.3)
Stool screening-treatment*		
Stool screened	350 (29.2)	–
Women with >1 ova-parasite identified	109/350 (31.0)	–
Women treated	84/109 (77.1)	–
Urinary tract infection (UTI)†		
Urine culture screen completed	605	–
UTI‡ present	21/605 (3.5)	–
Treated for UTI	16/21 (76.2)	–
Urine culture test of cure	20	–
No bacterial growth	20/20 (100)	–
Sexual transmitted/reproductive tract infections		
Gonorrhoea/Chlamydia testing done§, n	608	–
Gonorrhoea cases	0/608 (0)	–
Chlamydia cases	2/608 (0.3)	–
Chlamydia treated	2/2 (100)	–
Bacterial vaginosis (BV)/trichomonas testing done (symptomatic mothers)**¶**	40	–
Symptomatic trichomonas infection	1 (0.2)	–
Treated for symptomatic trichomonas	1 (100)	–
Symptomatic BV infection	4 (0.8)	–
Treated for symptomatic BV	2 (50)	–

All numbers are n (%) or n/N (%).

* Stool screening done from 31 May 2021 to December 2021.

† UTI screening/culture done from 3 August 2020 to 1 April 2021.

‡ UTI is defined as high burden growth of single organism (>100 k colony forming units (CFU)/mL) or significant growth (10 to <100 k CFU/mL) in symptomatic woman.

§ Gonorrhoea/Chlamydia screening and treatment done from 3 August 2020 to 1 April 2021.

¶ Bacterial vaginosis and trichomonas screening of women with vaginal symptoms was done from 5 October 2020 to 1 April 2021 (n=450 women enrolled/eligible for potential screening during the time period). Prevalence is reported among those eligible during the screening period.

EIMP, enhanced infection management package; ENAT, Enhancing Nutrition and Antenatal infection Treatment.

### Primary outcomes

Newborn weight was measured for 1990 infants (n=1610 <72 hours). Newborn length was measured for 1853 (n=1548 <72 hours). Infants who were not assessed were born to women who were younger, nulliparous, had lower nutritional status (BMI<18.5 kg/m^2^ or MUAC<23 cm), lived further from study health centres, or gave birth at home (online supplemental table S8). The mean (±SD) birth weight was 2877±451 g in the ENP arm and 2899±438 g in the routine nutrition care arm, with an adjusted mean difference (aMD) of −4 g (97.5% CI −83 to 75 g) (table 6). The mean birth length was 47.8±2.9 cm in the ENP and 48.3±2.8 cm in the routine nutrition care (aMD −0.31, 97.5% CI −1.1 to 0.49). The mean birth weight in the EIMP arm was 2893±435 g and in the routine infection care arm was 2881±455 g (aMD 18, 97.5% CI −35 to 70) (table 7). The mean birth length was 48.1±2.9 cm in the EIMP and 48.0±2.9 cm in the routine infection care (aMD 0.16, 97.5% CI −0.13 to 0.45). Cluster-specific primary outcome data are shown in online supplemental table S9–S10. There was no effect of the combined package (ENP+EIMP) compared with routine care on the primary outcomes (table 8). In sensitivity analysis including imputation of missing outcomes or birth weight and length measured >72 hours, the results were similar to the primary analysis, and we did not find an intervention effect on infant weight or length (online supplemental tables S11–S13).

**Table 6:** Effects of Enhanced Nutrition Package (ENP) on pregnancy outcomes

	InterventionENP*	Controlnot-ENP*	Intervention effect†, unadjusted (95% CI‡)	Intervention effect†, adjusted§ (95% CI‡)
Total known pregnancy outcomes (n)	1114	1056	–	–
Live births (n)	1089	1025	–	–
Primary outcomes				
Newborn weight (<72 hours)¶, mean (SD), g	2877 (451)	2899 (438)	−23 (−109, 63)	−4 (−83, 75)
Newborn length (<72 hours)**, mean (SD), cm	47.8 (2.9)	48.3 (2.8)	−0.36 (-1.15, 0.44)	−0.31 (-1.10, 0.49)
Secondary outcomes				
Gestational age††, mean (SD), wks	39.3 (4.3)	39.1 (4.2)	0.16 (-0.38, 0.71)	0.12 (-0.40, 0.64)
Preterm deliveries, n/N (%)	106/1114 (10.3%)	117/1056 (11.1%)	0.81 (0.47, 1.39)	0.81 (0.51, 1.28)
Preterm livebirths, n/N (%)	84/1085 (7.7%)	90/1017 (8.8%)	0.80 (0.46, 1.39)	0.80 (0.49, 1.30)
Small for gestational age, n/N (%)	324/861 (37.7%)	259/749 (34.6%)	1.16 (0.86, 1.55)	1.12 (0.82, 1.53)
Low birth weight (<2500 g), n/N (%)	137/861 (15.9%)	120/749 (16.0%)	0.90 (0.59, 1.38)	0.86 (0.56, 1.31)
Stillbirths, n/N (rate per 1000 births)	16/1105 (14.5)	24/1049 (22.9)	0.67 (0.29, 1.54)	0.67 (0.30, 1.53)
Newborn weight for age z-score¶, mean (SD)	−1.02 (0.96)	−0.91 (0.98)	−0.17 (-0.44, 0.11)	−0.13 (-0.42, 0.15)
Newborn length for age z-score**, mean (SD)	−0.91 (1.42)	−0.60 (1.38)	−0.31 (-0.61, 0.00)	−0.29 (-0.61, 0.03)
Newborn head circumference‡‡, mean (SD), cm	34.6 (1.7)	34.4 (1.7)	0.2 (-0.3, 0.7)	0.3 (-0.1, 0.7)
Maternal anaemia (Hb§§<110 g/L), n/N (%)	136/332 (40.8%)	183/405 (45.2%)	0.80 (0.26, 2.43)	0.77 (0.26, 2.35)

* Descriptive statistics are reported at the individual level for each study group.

† Cluster level mean differences are shown for continuous outcomes, and relative risks are shown for dichotomous outcomes.

‡ For the primary outcomes, 97.5% CIs are reported adjusting for multiplicity (coprimary outcomes) using Bonferroni correction. For secondary outcomes, 95% CIs were not adjusted for multiplicity and should not be used in place of hypothesis testing.

§ Adjusted for a priori prognostic factors and imbalanced variables: maternal parity, BMI at baseline, height, education and occupation.

¶ Among livebirths, weight was measured within <72 hours of life for n=861 in ENP arm and n=749 for not-ENP arm.

** Among livebirths, length was measured within <72 hours of life for n=839 in ENP arm and n=709 for not-ENP arm.

†† Among pregnancy outcomes, gestational age was available for n=1110 in ENP arm and n=104 in not-ENP arm.

‡‡ Among livebirths, head circumference measured within <72 hrs of life for n=842 in the ENP arm and n=709 in the not-ENP arm.

§§ Hb adjusted for altitude (Hb adjustment (g/L)=(0.0056384×elevation)+(0.0000003×elevation2); Hb available in n=332 in ENP arm and n=405 in not-ENP arm.

BMI, body mass index; ENP, enhanced nutrition package; Hb, haemoglobin.

**Table 7:** Effects of Enhanced Infection Management Package (EIMP) on pregnancy outcomes

	InterventionEIMP*	Controlnot-EIMP*	Intervention effect†, unadjusted (95% CI‡)	Intervention effect†, adjusted§ (95% CI‡)
Total known pregnancy outcomes (n)	1090	1080	–	–
Live births (n)	1070	1044	–	–
Primary outcomes				
Newborn weight (<72 hours)¶, mean (SD), g	2893 (435)	2881 (455)	13 (-46, 72)	18 (-35, 70)
Newborn length (<72 hours)**, mean (SD), cm	48.1 (2.9)	48.0 (2.9)	0.16 (-0.13, 0.46)	0.16 (-0.13, 0.45)
Secondary outcomes				
Gestational age††, mean (SD), wks	39.3 (4.2)	39.2 (4.3)	0.06 (-0.29, 0.42)	0.08 (-0.29, 0.46)
Proportion preterm deliveries, n (%)	115/1090 (10.6%)	108/1080 (10%)	1.09 (0.83, 1.44)	1.04 (0.80, 1.36)
Preterm live birth prevalence, n (%)	93/1065 (8.7%)	81/1037 (7.8%)	1.12 (0.86, 1.46)	1.08 (0.82, 1.41)
Small for gestational age, n (%)	298/796 (37.4%)	285/814 (35.0%)	1.07 (0.94, 1.22)	1.07 (0.93, 1.22)
Low birth weight (<2500 g), n (%)	131/796 (16.5%)	126/814 (15.5%)	1.06 (0.96, 1.18)	1.05 (0.94, 1.16)
Stillbirth rate, n (per 1000 births)	15/1085 (13.8)	25/1069 (23.4)	0.59 (0.27, 1.28)	0.59 (0.27,1.33)
Newborn weight for age z-score¶, mean (SD)	−0.98 (0.97)	−0.96 (0.97)	−0.02 (-0.12, 0.07)	−0.02 (-0.11, 0.07)
Newborn length for age z-score**, mean (SD)	−0.76 (1.4)	−0.78 (1.4)	0.02 (-0.08, 0.12)	0.01 (-0.09, 0.10)
Newborn head circumference (<72 hours)‡‡, mean (SD), cm	34.6 (1.7)	34.5 (1.7)	0.14 (−0.01, 0.29)	0.14 (-0.02, 0.30)
Maternal anaemia (Hb§§ <110 g/L), n/N (%)	169/385 (43.8%)	150/352 (42.6%)	1.03 (0.93, 1.13)	1.02 (0.93, 1.12)

* Descriptive statistics are reported at the individual level for each study group.

† Mean differences are shown for continuous outcomes, and relative risks are shown for dichotomous outcomes.

‡ For the primary outcomes, 97.5% CIs are reported adjusting for multiplicity (coprimary outcomes) using Bonferroni correction. For secondary outcomes, 95% CIs were not adjusted for multiplicity and should not be used in place of hypothesis testing.

§ Adjusted for a priori prognostic factors and imbalanced variables: maternal parity, BMI at baseline, height, education and occupation.

¶ Among livebirths, weight was measured within <72 hours of life for n=796 in EIMP arm and n=814 in not-EIMP arm.

** Among livebirths, length was measured within<72 hours of life for n=762 in EIMP arm and n=786 in not-EIMP arm.

†† Among pregnancy outcomes, gestational age was available in n=1084 in EIMP arm and n=1073 in not-EIMP arm.

‡‡ Among livebirths, head circumference was measured within <72 hours of life for n=764 in EIMP arm and n=787 in not-EIMP arm.

§§ Altitude adjusted Hb (Hb adjustment (g/L)=(0.0056384×elevation)+(0.0000003×elevation2); Hb available in n=385 in EIMP arm and n=352 in not-EIMP arm.

BMI, body mass index; EIMP, enhanced infection management package; Hb, haemoglobin.

**Table 8:** Effects of ENP+EIMP (vs neither intervention) on pregnancy outcomes

	ENP+EIMP*	Neither intervention*	Intervention effect†, unadjusted (95% CI‡)	Intervention effect†, adjusted§ (95% CI‡)
Total known pregnancy outcomes (n)	565	531	–	–
Live births (n)	559	514	–	–
Primary outcomes				
Newborn weight¶, mean (SD), g	2881 (443)	2890 (450)	−15 (−120, 91)	14 (-81, 109)
Newborn length**, mean (SD), cm	47.9 (3.0)	48.1 (3)	−0.1 (-1.3, 1.0)	−0.1 (-1.2, 1.1)
Secondary outcomes				
Gestational age††, mean (SD), weeks	39.3 (4.4)	39.1 (4.4)	0.23 (-0.29, 0.74)	0.23 (-0.30, 0.76)
Proportion preterm deliveries, n (%)	57/565 (10.1%)	59/531 (11.1)	0.97 (0.61, 1.53)	0.91 (0.57, 1.45)
Preterm live birth prevalence, n (%)	48/557 (8.6%)	45/509 (8.8%)	0.97 (0.61, 1.55)	0.91 (0.55, 1.49)
Small for gestational age, n (%)	171/426 (40.3)	132/379 (34.8)	1.32 (0.80, 2.19)	1.26 (0.76, 2.10)
Low birth weight (<2500 g), n (%)	71/426 (16.7)	60/379 (15.8)	0.99 (0.61, 1.61)	0.92 (0.56, 1.50)
Stillbirth rate, (per 1000 births)	4/563 (7.1)	13/527 (24.7)	0.29 (0.08, 1.06)	**0.29 (0.09, 0.94)**
Newborn weight¶ for age z-score, mean (SD)	−1.05 (0.96)	−0.92 (0.97)	−0.24 (-0.61, 0.13)	−0.19 (-0.56, 0.18)
Newborn length** for age z-score, mean (SD)	−0.93 (1.46)	−0.64 (1.38)	−0.28 (-0.61, 0.04)	−0.27 (-0.60, 0.07)
Newborn head circumference‡‡, mean (SD)	34.7 (1.7)	34.3 (1.7)	0.35 (-0.17, 0.88)	0.43 (-0.03, 0.89)
Maternal anaemia (Hb§§ <110 g/L), n/N (%)	73/175 (41.5%)	87/195 (44.6%)	0.93 (0.56, 1.55)	0.92 (0.56, 1.50)

Bold Values are statistically significant

* Descriptive statistics are reported at the individual level for each study group.

† Mean differences are shown for continuous outcomes, and relative risks are shown for dichotomous outcomes.

‡ For the primary outcomes, 97.5% CIs are reported adjusting for multiplicity (coprimary outcomes) using Bonferroni correction. For secondary outcomes, 95% CIs were not adjusted for multiplicity and should not be used in place of hypothesis testing.

§ Adjusted for a priori prognostic factors and imbalanced variables: maternal parity, BMI at baseline, height, education, occupation.

¶ Among livebirths, birth weight was measured within <72 hours for 426 for ENP+EIMP arm and 379 in routine care arm.

** Among livebirths, length was measured within <72 hours for 413 in ENP+EIMP arm and 360 in routine care arm.

†† Among pregnancy outcomes, gestational age was available for 563 in ENP+EIMP arm and 526 routine care arm.

‡‡ Head circumference available for 41 in ENP+EIMP arm and 361 in routine care arm

§§ Altitude adjusted Hb (Hb adjustment (g/L)=(0.0056384×elevation)+(0.0000003×elevation2); Hb available in n=175 in ENP+EIMP arm and n=195 in neither intervention arm.

BMI, body mass index; EIMP, enhanced infection management package; ENP, enhanced nutrition package; Hb, haemoglobin.

### Secondary outcomes

Women in the ENP study arm had lower prevalence of preterm delivery, preterm live birth, LBW, stillbirth and anaemia compared with women in routine nutrition care health centres although statistical evidence for any difference was weak (table 4a). The EIMP effects on secondary outcomes were also null (table 4b). For the comparison of women receiving both ENP and EIMP packages to those receiving neither package, the stillbirth rate was significantly lower among women receiving both interventions (ENP+EIMP: 7.1/1000 births vs neither: 24.7/1000 births; adjusted relative risk 0.29, 95% CI 0.09 to 0.94) (table 4c), primarily due to reduction in stillbirths <37 weeks gestation. In the ENP+EIMP group, the majority (75%) of stillbirths were full-term stillbirths (>37 wk), which are more likely intrapartum-related, compared with the routine care group, in which 54% of stillbirths were full term. Head circumference was slightly larger in the group receiving both intervention packages, though not at a level of significance (aMD 0.43 cm, 95% CI −0.03 to 0.89).

### Secondary and subgroup analyses

The subgroup analysis of women who ever had MUAC<23 cm (ie, those targeted to receive BEP supplementation) was limited by missing data and differential follow-up. We found no between-group differences (ENP vs routine nutrition care) for the primary outcomes of birth weight or length (online supplemental table S14) in analysis with imputation of missing outcomes. We also found no effects on birth size in per-protocol analysis among women with higher BEP adherence (>50% of eligible days).

In a priori subgroup analysis, we explored effect modification by maternal BMI, age and parity and did not find significant differences in intervention effects by these subgroups (online supplemental table S15).

There were also no differences in major maternal morbidities (eclampsia, pre-eclampsia, gestational diabetes, malaria, tuberculosis, HIV) across study arms (online supplemental table S16).

## Discussion

In our pragmatic effectiveness study, the implementation of ANC packages including WHO-recommended interventions for maternal nutrition and infection management delivered within the Ethiopian health system did not impact the primary study outcomes of newborn birth weight or length. The intervention packages increased demand for ANC care, with a higher number of pregnancy ANC contacts and coverage of four ANC contacts in intervention arms. One in three women in the nutrition arm was eligible for BEP supplementation; however, average adherence to the supplement was lower than anticipated at half of the eligible days. The prevalence of genitourinary tract infections was also very low (~5%), although parasitic stool infections affected one in three women. The rate of stillbirth was lower in the combined nutrition and infection intervention group compared with women receiving neither intervention package.

We did not detect an impact of BEP supplementation on newborn birth size in our effectiveness study, a contrast to findings from recent efficacy trials and meta-analyses. In a 2015 Cochrane meta-analysis,^48^ BEP supplementation increased mean birth weight 41 g (11 trials; 95% CI 5 to 77 g), with greater effects in under-nourished pregnant women (8 trials; 67 g, 95% CI 12 to 121 g), while there were no effects on birth length. The recent MISAME (MIcronutriments pour la SAnté de la Mère et de l'Enfant) efficacy trial in Burkina Faso used an energy-dense peanut paste BEP to supplement pregnant women and reported higher birth weight (49.7 g) and length (0.2 cm) in the BEP group.^47^ There are several potential explanations for the lack of impact in our study. First, the study arms were imbalanced at baseline, with ENP clusters having higher baseline rates of maternal undernutrition (BMI<18.5 kg/m^2^) and being of greater socioeconomic disadvantage (lower education and more agricultural labour). It is possible that we were not able to adequately adjust for this imbalance, and that the results were limited by the small number of clusters. Second, the adherence to BEP supplements may have been inadequate, with an average of 74 days of consumption and only half of women consuming BEP >50% of eligible days. BEP distribution and adherence were affected by COVID-19 and the local security situation. Although the BEP was a vegan product, consumption may have been affected by long hours of fasting, typically from 20:00 to 15:00 the next day, and skipping meals is common in this orthodox Christian community.^26^ Another potential explanation for the lack of effect may be the relatively later initiation of the intervention during gestation (second trimester). The WINGs and Women First trial demonstrated the beneficial effects of earlier supplementation in the first trimester and preconception.^49 50^ Finally, it is possible that the BEP supplement may potentially have displaced normal food intake. Although women were counselled to treat the BEP as a supplement, it is possible that the BEP served as a primary food source, particularly during a time of food insecurity.

The infection package alone did not affect primary or secondary outcomes in our study, likely due to the very low prevalence of genitourinary tract infections and inadequate treatment of intestinal parasitic infections. In a recent systematic review,^51^ screening and treatment of asymptomatic bacteriuria was identified as a promising intervention to prevent LBW and preterm birth; however, the quality of evidence was graded as low. The prevalence of UTI in our study (3.5%) was lower than anticipated. In a recent systematic review, the pooled prevalence of UTI in pregnancy in Ethiopia was 15.4% (95% CI 12.5% to 18.2%).^52^ Gonorrhoea and chlamydia testing was done using sensitive lab methods; however, the prevalence was <1% in this rural community. Intestinal parasitic infections had a higher prevalence in the study population, however, with a predominance of waterborne infections (Giardia and Amoeba) that were not treated by presumptive mebendazole. The pathogen-specific treatment in the third trimester may have been too late to affect fetal growth or inflammation predisposing to preterm birth.

Undernutrition and infections in pregnancy may interact to adversely affect pregnancy outcomes. We aimed to intervene on both domains simultaneously to achieve greater benefits on fetal growth and pregnancy duration, though our combined packages did not impact our primary outcome of birth size. The combined intervention arm had lower rates of stillbirth compared with the routine care arm, and that was primarily a reduction in stillbirths <37 weeks gestation. Stillbirth was a predefined secondary outcome that was not adjusted for multiple comparisons; thus, it is also possible the finding could be due to chance. This hypothesis-generating finding should be supported by additional studies. Fetal growth restriction is the single largest risk factor for stillbirth,^53^ and in a 2020 Cochrane review, prenatal BEP supplementation reduced stillbirth risk by 40% (5 RCTs, RR 0.60; 95% CI 0.39 to 0.94).^54^ Maternal infections also place infants at risk of stillbirth,^55 56^ and the infection arm had a similar magnitude of rate reduction, with both interventions having at least additive effects on stillbirth. Other trials have tested the impact of combined nutrition and infection interventions and report varying results on birth outcomes. In a cluster RCT implemented in 22 neighbouring health centres Amhara and Oromia, Ethiopia,^57^ a health centre level intervention bundle strengthened basic ANC service provision, including screening for maternal infection (urine dipstick, syphilis point of care testing) and anaemia, and resulted in higher birth weight (108 g, 95% CI 91 to 125 g). However, the study design and interventions were different from the current study. In a randomised trial of maternal nutrition and infection interventions in Sierra Leone,^58^ undernourished women were randomised to receive a ready-to-use food supplement, presumptive azithromycin, intermittent preventive treatment of malaria in pregnancy, and treatment for vaginal dysbiosis, versus standard of care. Infants in the intervention arm were 70 g heavier (95% CI 20 to 120) and 0.3 cm longer (95% CI 0.09 to 0.6) than the control arm, although there was no difference in stillbirth rates. The WINGS study in India^50^ implemented an integrated package including food supplements, genitourinary tract infection treatment, and water, sanitation and hygiene interventions. Interventions in the pregnancy period did not affect birth weight, length or stillbirth, but SGA prevalence was lower in the intervention arm. The most prominent effects were found for interventions delivered in both the preconception and pregnancy periods.

Our interventions positively influenced ANC care-seeking, increasing coverage of four ANC contacts more than twofold. Based on patient feedback, the iodised salt supply was a demand incentive for attending ANC. In a 2015 Cochrane review, single health system or community interventions resulted in only marginal improvements in four ANC visit coverage (OR 1.11, 95% CI 1.01 to 1.22).^59^ The ENAT strategy shows that the provision of nutritional supplements within ANC visits significantly increased coverage during a challenging period and may be a strategy to increase ANC coverage, particularly with the new WHO recommendations.

There were several strengths of our study. We engaged local stakeholders and community members in the co-design of the intervention packages to maximise uptake and acceptability,^27^ which was critical in the selection of a well-liked, vegan and locally produced BEP product. Interventions were delivered by health system staff to test the effectiveness of pragmatic, real-world implementation. All study outcome measurements were rigorous and done by research staff. We conducted extensive training, standardisation and quality control of neonatal anthropometrics^35^ and ultrasound measurement, resulting in high quality of birth weight and GA data. We had high follow-up of pregnancy outcome status and had high quality and minimal missing GA data.

Limitations in our study resulted from the constrained field access and follow-up visits during the study period due to the pandemic and security situation. These conditions affected BEP distribution and adherence, as well as timing of study visits for home births. We also faced imbalanced follow-up rates due to higher ANC attendance in the intervention arms. The differential missingness of outcome measurements may have influenced our findings, and we conducted extensive sensitivity analyses to address missing data, including multiple imputation to assess potential bias.^60^ This sensitivity analysis, including missing measures, did not influence our main findings.

## Conclusions

In rural Amhara, the pragmatic delivery of integrated ANC packages of enhanced nutrition support and infection control within the Ethiopian health system did not improve fetal growth, likely due to the relatively low BEP supplement adherence and low prevalence of genitourinary tract infections. However, stillbirth rates were lower in the combined infection and nutrition intervention arm compared with routine care. We also found that improving the quality of services significantly increased ANC care-seeking and coverage. These findings emphasise the importance of future research to address implementation barriers to achieve effective intervention coverage within different health systems and contexts, as well as the need for intensified efforts to develop new interventions and approaches to prevent small vulnerable birth outcomes.

## Supplementary material

10.1136/bmjgh-2024-016264 online supplemental file 1

10.1136/bmjgh-2024-016264 online supplemental file 2

## Data Availability

Data are available on reasonable request.
