# Supplementary material for: The impact of enhancing nutrition and antenatal infection treatment on birth outcomes in Amhara, Ethiopia: a pragmatic factorial, cluster-randomised clinical effectiveness study
Source: BMJ Glob Health. 2025 Jun 18;10(6):e016264. doi: 10.1136/bmjgh-2024-016264 (PMC12181995; doi:10.1136/bmjgh-2024-016264)
Supplement: online supplemental file 1 [file bmjgh-10-6-s001.pdf]

BMJ Paediatrics Open

Enhancing Nutrition and Antenatal Infection Treatment (ENAT) study: protocol of a pragmatic clinical effectiveness study to improve birth outcomes in Ethiopia

Anne CC Lee ,[1,2] Firehiwot Workneh Abate,[3] Luke C Mullany,[4] Estifanos Baye,[1,2] Yoseph Yemane Berhane,[3] Mulatu Melese Derebe,[5] Michelle Eglovitch,[1] Nebiyou Fasil,[3] Ingrid E Olson,[1] Workagegnehu Tarekegn Kidane,[3] Tigest Shiferaw,[3] Fisseha Shiferie,[3] Fitsum Tsegaye,[3] Sitota Tsegaye,[3] Kalkidan Yibeltal,[3] Grace J Chan ,[2,6,7] Parul Christian,[4] Sheila Isanaka,[8] Yunhee Kang,[4] Chunling Lu,[2,9] Mandefro M Mengistie,[10] Rose L. Molina,[2,11] Michele D Stojanov,[11] Fred Van Dyk,[4] Amare Worku Tadesse,[3,12] Asresie T Wondale,[13] Blair J Wylie,[2,11] Alemayehu Worku,[3] Yemane Berhane[3]

To cite: Lee ACC, Abate FW, Mullany LC, *et al*. Enhancing Nutrition and Antenatal Infection Treatment (ENAT) study: protocol of a pragmatic clinical effectiveness study to improve birth outcomes in Ethiopia. *BMJ Paediatrics Open* 2022;**6**:e001327. doi:10.1136/bmjpo-2021-001327

► Additional supplemental material is published online only. To view, please visit the journal online (http://dx.doi.org/10.1136/bmjpo-2021-001327).

Received 21 October 2021
Accepted 7 November 2021

Check for updates

© Author(s) (or their employer(s)) 2022. Re-use permitted under CC BY. Published by BMJ.

For numbered affiliations see end of article.

Correspondence to
Dr Anne CC Lee; alee6@bwh.harvard.edu

Professor Yemane Berhane; yemaneberhane@addiscontinental.edu.et

ABSTRACT

Introduction The WHO Nutrition Target aims to reduce the global prevalence of low birth weight by 30% by the year 2025. The Enhancing Nutrition and Antenatal Infection Treatment (ENAT) study will test the impact of packages of pregnancy interventions to enhance maternal nutrition and infection management on birth outcomes in rural Ethiopia.

Methods and analysis ENAT is a pragmatic, open-label, 2×2 factorial, randomised clinical effectiveness study implemented in 12 rural health centres in Amhara, Ethiopia. Eligible pregnant women presenting at antenatal care (ANC) visits at <24 weeks gestation are enrolled (n=2400). ANC quality is strengthened across all centres. Health centres are randomised to receive an enhanced nutrition package (ENP) or standard nutrition care, and within each health centre, individual women are randomised to receive an enhanced infection management package (EIMP) or standard infection care. At ENP centres, women receive a regular supply of adequately iodised salt and iron–folate (IFA), enhanced nutrition counselling and those with mid-upper arm circumference of <23 cm receive a micronutrient fortified balanced energy protein supplement (corn soya blend) until delivery. In standard nutrition centres, women receive routine counselling and IFA. EIMP women have additional screening/treatment for urinary and sexual/reproductive tract infections and intensive deworming. Non-EIMP women are managed syndromically per Ministry of Health Guidelines. Participants are followed until 1-month post partum, and a subset until 6 months. The primary study outcomes are newborn weight and length measured at <72 hours of age. Secondary outcomes include preterm birth, low birth weight and stillbirth rates; newborn head circumference; infant weight and length for age z-scores at birth; maternal anaemia; and weight gain during pregnancy.

Ethics and dissemination ENAT is approved by the Institutional Review Boards of Addis Continental Institute of Public Health (001-A1-2019) and Mass

What is already known on this topic?

- ► In low-income and middle-income countries, maternal undernutrition is prevalent and a major risk factor for adverse birth outcomes, including spontaneous preterm birth, low birth weight and small for gestational age infants.
- ► Maternal infections in pregnancy are also common, yet under-recognised risk factors for preterm birth and poor fetal growth in low-income and middle-income countries.
- ► Beyond their independent effects, maternal infections and nutritional status may have synergistic effects on fetal growth and gestational length.

What this study hopes to add?

- ► Increase the evidence-base on the role of antenatal infection management on maternal and birth outcomes in a low resource rural setting in sub-Saharan Africa.
- ► Increase the evidence-base on the role of targeted, fortified balanced energy protein supplementation on maternal and birth outcomes in a low resource rural setting in sub-Saharan Africa.
- ► Evaluate the benefit of developing intervention packages with local stakeholders that are implemented within existing health systems.

bmjpo: first published as 10.1136/bmjpo-2021-001327 on 13 January 2022. Downloaded from http://bmjpaedsopen.bmj.com/ on April 25, 2024 by guest. Protected by copyright.

General Brigham (2018P002479). Results will be disseminated to local and international stakeholders.
Registration number ISRCTN15116516.

INTRODUCTION

The WHO Third Global Nutrition Target aims to reduce the proportion of babies born with low birth weight (LBW,<2500 g) by 30% by the year 2025.[1] Approximately 20.5 million infants were born LBW in 2015, with 91% from low-income and middle-income countries (LMIC) and 24% in sub-Saharan Africa.[2] The main aetiologies of LBW are preterm birth (<37 gestational weeks) and fetal growth restriction, commonly classified as small-for-gestational-age (SGA) at birth. Preterm and SGA infants carry increased risk of mortality, morbidity, childhood stunting, neurodevelopmental impairment and adult chronic disease.[3–7] Prevention of preterm birth and SGA is a key public health strategy to improve child survival and health in LMICs.

In LMICs, maternal undernutrition is prevalent and a major risk factor for adverse birth outcomes, including spontaneous preterm birth, LBW and SGA.[8 9] Interventions to improve maternal nutritional status in pregnancy, including iron–folate (IFA),[10] multiple micronutrients[11] and balanced protein-energy (BEP) supplementation,[12 13] have been individually tested and found to be effective in increasing mean birth weight. However, lower than expected benefit has been observed with the individual nutritional interventions.[10 12–14] Greater effect sizes are noted in undernourished women, and there is a need for additional studies with standardised supplementation criteria as well as the combination of BEP and micronutrient supplementation.[12 13]

Maternal infections in pregnancy are also common, yet under-recognised risk factors for preterm birth and poor fetal growth in LMICs. Urinary tract infection or asymptomatic bacteriuria may affect 9%–80% of pregnancies in sub-Saharan Africa[15] and are associated with a twofold elevated risk of preterm delivery.[16] Helminthic infections are prevalent and associated with systemic inflammation, LBW and preterm birth.[17–19] Genital tract infections may ascend the reproductive tract and lead to infection and inflammation in the amniotic fluid, predisposing to preterm birth.[20] In LMICs, screening and treatment of genitourinary tract infections during routine antenatal care (ANC) is infrequent due to lack of resources and capacity for laboratory testing. While epidemiological data has consistently established associations between prenatal infections and adverse pregnancy outcomes, there is limited evidence on the effectiveness of prenatal interventions to screen and treat infections to prevent LBW in LMICs.

Beyond their independent effects, maternal infections and nutritional status may have synergistic effects on fetal growth and gestational length.[21–25] Figure 1 depicts several pathways linking maternal nutrition and infections in pregnancy. This framework provides the basis for the hypothesis that targeting both risk factors in pregnancy may lead to more substantial, and potentially synergistic, improvements in fetal growth and pregnancy length.[26]

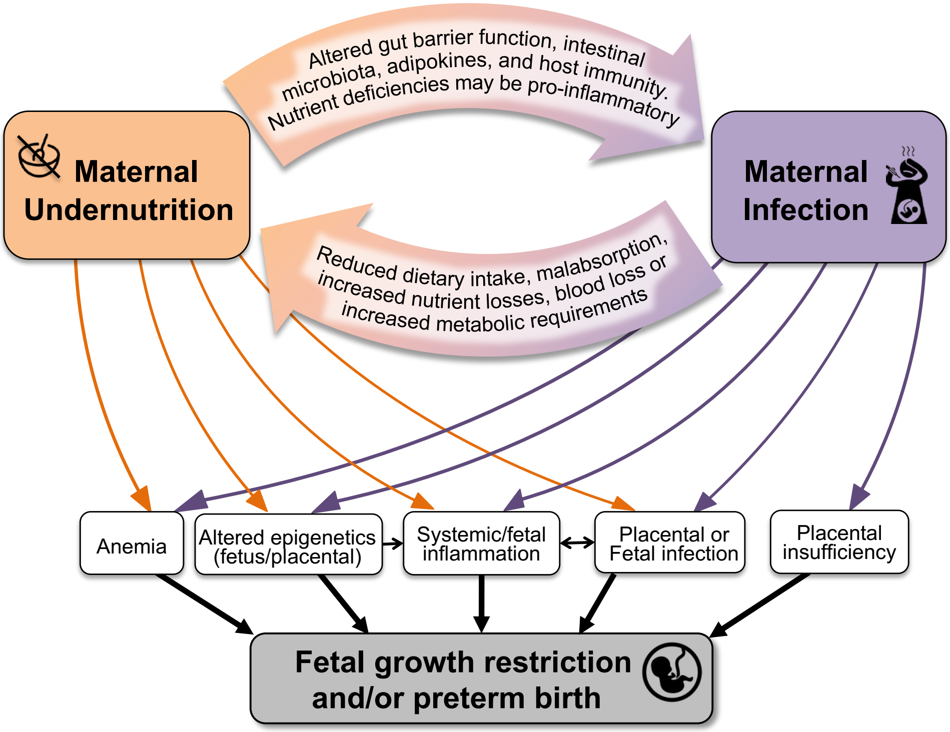

Figure 1 Conceptual diagram showing the pathways that link maternal undernutrition, maternal infection and infant outcomes .

bmjpo: first published as 10.1136/bmjpo-2021-001327 on 13 January 2022. Downloaded from http://bmjpaedsopen.bmj.com/ on April 25, 2024 by guest. Protected by copyright.

This manuscript details the study protocol for the Enhancing Nutrition and Antenatal Infection Treatment (ENAT) study in Amhara region, Ethiopia. In 2016, WHO released recommendations on an evidence-based, core package of ANC to optimise the pregnancy experience and outcomes,[27] include guidelines on nutrition and infection management in pregnancy (online supplemental web table 1). In Ethiopia, not all recommendations have been adopted or achieved high level of coverage or quality in ANC. The primary aim of the ENAT study is to determine the impact of certain WHO-recommended ANC interventions to optimise maternal nutrition and manage maternal pregnancy infections on infant birth size in Amhara, Ethiopia. We hypothesise that the nutrition and infection management packages will independently increase newborn birth weight and length, and that the combined effect of both packages will be greater than either alone.

METHODS AND ANALYSIS

Study design

The ENAT study is a 2×2 factorial pragmatic, open label, randomised clinical effectiveness study with cluster randomisation of the enhanced nutrition package (ENP) versus standard care (non-ENP) and individual level randomisation of an enhanced infection management package (EIMP) versus standard care (non-EIMP) (figure 2).

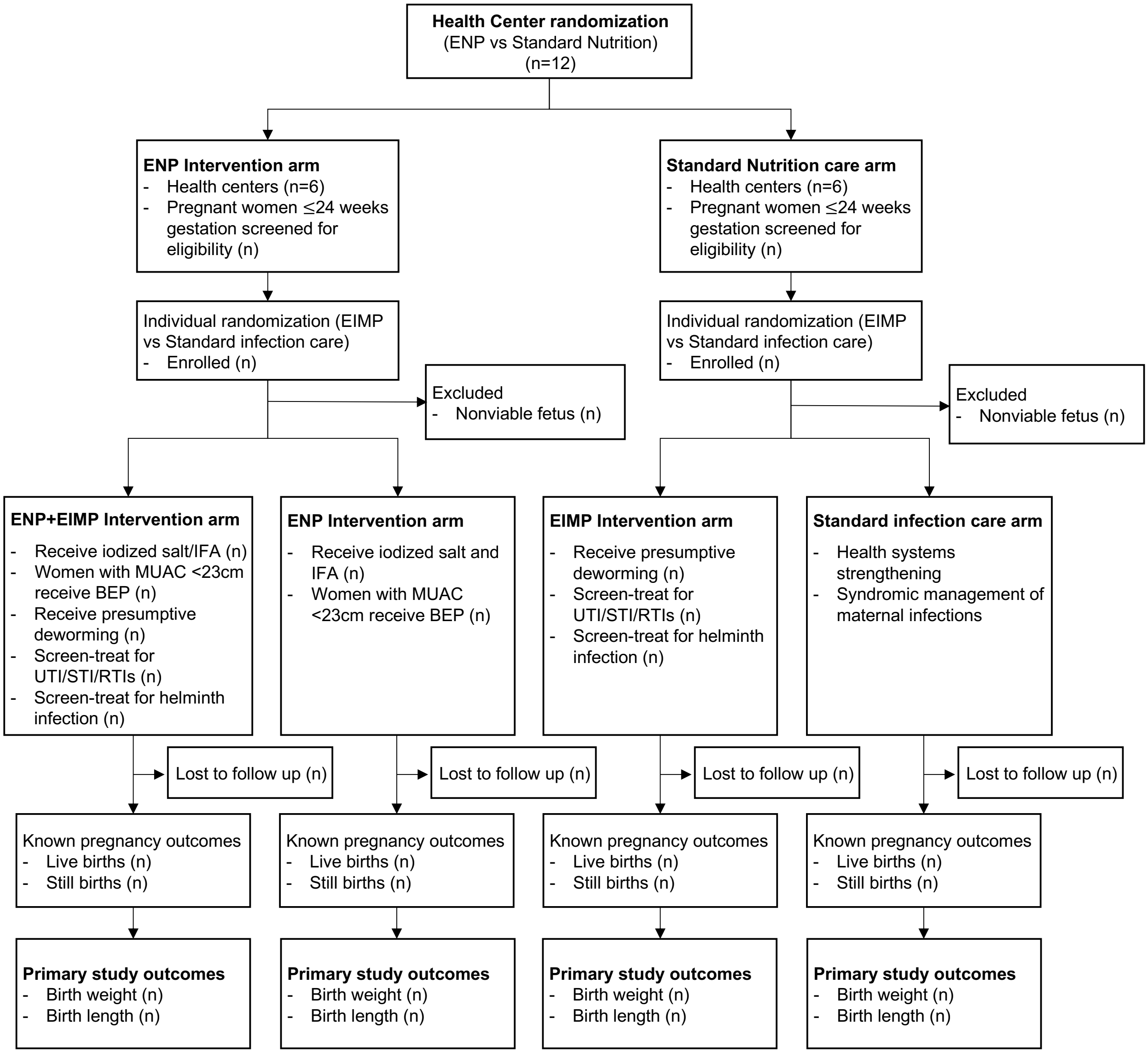

Figure 2 Enhancing Nutrition and Antenatal Infection Treatment study consort diagram. BEP, balanced protein-energy; EIMP, enhanced infection management package; ENP, enhanced nutrition package; GA, gestational age; IFA, iron–folate; MUAC, mid-upper arm circumferences; ; RTI, reproductive tract infection; STI, sexually transmitted infection; UTI, urinary tract infection.

bmjpo: first published as 10.1136/bmjpo-2021-001327 on 13 January 2022. Downloaded from http://bmjpaedsopen.bmj.com/ on April 25, 2024 by guest. Protected by copyright.

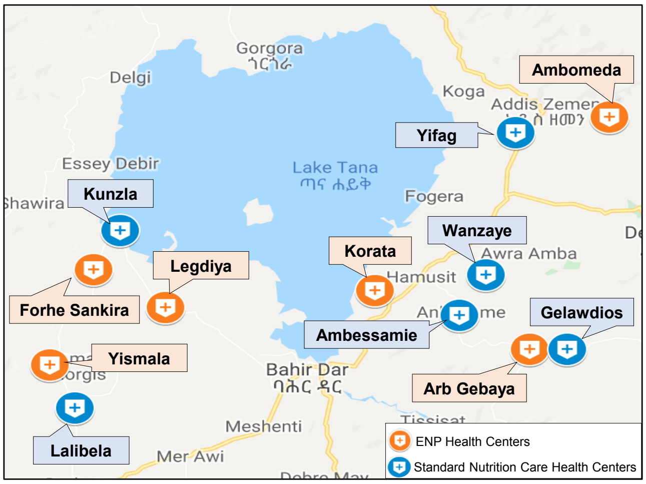

Figure 3 Enhancing Nutrition and Antenatal Infection Treatment study site map, Amhara region, Ethiopia. ENP, enhanced nutrition package.

Study setting

The ENAT study site was established in 2018 as a partnership with the Addis Continental Institute of Public Health (ACIPH), Amhara Regional Health Bureau (ARHB), Amhara Public Health Institute (APHI) and Brigham and Women's Hospital. The Amhara region has low-resourced health systems and poor health indicators. As per the 2016 Ethiopian Demographic Health Services data, Amhara had the country's highest rates of neonatal mortality (47 per 1000 live births) and LBW (22.2%), and high maternal mortality rate (412 per 100 000 live births).[28] Rates of any prenatal care and institutional delivery were 82.6% and 54.2%, respectively.[29] One in four women of reproductive age are underweight (body mass index (BMI) <18.5 kg/m^2)[28] and geohelminth infections are prevalent, ranging from 21.1% to 43.5%.[30 31]

The ENAT study is conducted in 12 rural health centres (each serving~25 000 population) in West Gojjam (South and North Achefer districts (woredas)) and South Gondar (Dera and Libokemem districts) zones, Amhara (figure 3, Site Map). The districts were chosen in collaboration with the ARHB based on the high rates of undernutrition, risk of LBW, need for nutritional programmes and proximity to the regional laboratory. The study health centres were chosen based on accessibility, total ANC volume (minimum 250 women presenting to ANC/year) and infrastructure (functional laboratory).

Patient and public involvement

Prior to the study, formative work was conducted with a range of community members (mothers, families, community and religious leaders, health providers).[32] This feedback directly informed the design of the study interventions, packages and their implementation. Community sensitisation was performed prior to initiating the study.

Study participants and recruitment

Pregnant women are recruited from ANC visits in designated ENAT study health centres. A study nurse explains study procedures and obtains written informed consent. For illiterate women, an impartial witness attests to consent and the woman provides a thumbprint.

To encourage early presentation at ANC clinics, community sensitisation was conducted prior to study initiation. Study field data collectors and community cadres disseminated information about the ENAT study at monthly community-based pregnant women's conferences, and community and religious gatherings and encouraged presentation to the health centres if/as soon as pregnancy was suspected. Study enrolment began in August 2020 and will continue until the sample size has been met. As of 1 September 2021 ENAT has enrolled 2148 women.

Inclusion criteria

- ≤24 weeks gestation based on a clinical algorithm (last menstrual period and/or symphysis fundal height) who have a viable pregnancy.

Exclusion criteria

- Pregnant women presenting for first ANC >24 weeks.
- Pregnant women who live >2 hours walking distance from ENAT health centres.
- Pregnant women presenting at first ANC with fetus that is non-viable (without a heartbeat on enrolment ultrasound).

ENAT study interventions

Health systems strengthening of ANC services was performed in ENAT health centres prior to the study to benefit all women receiving care in these facilities. Health systems strengthening was conducted in partnership with ARHB and Jhpiego. Health center staff were trained in ANC standards, guidelines and measurements (blood pressure, gestational age, birth weight[33]). Facilities were stocked with basic equipment, medications and diagnostic testing. Laboratory capacity was augmented, and all health centres were equipped with ultrasound machines (GE Vscan Access, General Electric, Boston, MA). ENAT study interventions are delivered within the health system at routine ANC visits by health center staff, with supervision provided by research staff.

ENP

Nutritional counselling on adequate pregnancy nutrition and weight gain

Routine ANC nutrition counselling includes increasing intake and dietary diversity, however, can vary depending on patient load. For ENP centres, supplementary, locally contextualised, counselling material was developed based on our formative work,[32] and is delivered by midwives. Content includes BMI-based recommended weight gain, dietary diversity and educational messages developed to address local cultural beliefs related to dietary intake during pregnancy as well as side effects of IFA. Nutrition

bmjpo: first published as 10.1136/bmjpo-2021-001327 on 13 January 2022. Downloaded from http://bmjpaedsopen.bmj.com/ on April 25, 2024 by guest. Protected by copyright.

Table 1 Enhanced nutrition package (ENP) components

ENP content	Activity	Frequency
Nutritional education/ counselling	Counselling about healthy eating, adequate pregnancy weight gain, increasing protein and energy in diet, importance of iron/ folate, iodised salt.	Every ANC visit
Iron–folate	Strengthen counselling, supply and reinforcement of daily IFA (60 mg Fe/400 µg folic acid).	Provide initial supply per Ethiopian Federal Ministry of Health at enrollment; reinforce adherence, restock, and manage side effects at follow-up ANC visits.
Iodised salt	Provide supply (600 gm) of adequately iodized salt (30-40ppm) for routine household use during pregnancy; adherence monitoring and counselling.	Enrolment and monthly follow-up ANC visits
Balanced energy protein supplement	For pregnant women with MUAC <23 cm, provide locally-produced, micronutrient fortified, corn soya blend (SuperCereal) [200 g daily supplement (760 kcal/day, 28 g protein)].	Daily supplement, distributed at enrolment and follow-up ANC visits

ANC, antenatal care; ENP, enhanced nutrition package; IFA, iron–folate; MUAC, mid-upper arm circumferences.

education materials, including posters and videos, are shown in ENP health centres to promote women's behaviour change and maximise their exposure to various but consistent nutrition messages (table 1).

IFA

The Ethiopian Federal Ministry of Health (FMOH) recommends 60 mg iron plus 400 µg folic acid supplements, orally once daily in pregnancy. In August–September 2019, coverage of IFA was 46.3% in the ENAT health centres, and our formative work indicated that barriers included local beliefs about delivering 'big babies' and side effects such as constipation. In the ENP health centres, additional counselling is conducted, using video/media to address common cultural beliefs and side effects. Women are reminded at each ANC visit about IFA consumption, management of side effects and provided refills when their home IFA supply is low.

Provision of adequately iodised household salt

In ENP centres, we provide a monthly household supply of high-quality adequately iodised salt at every ANC visit. The iodised salt (Waff Manufacturing, 30–40 ppm potassium iodate, 600 g bottle) is packaged in airtight, resealable, polyethylene containers, to allow resealing after use and to reduce risk of evaporative losses at the household level. Quality control procedures are in place to ensure that iodisation is in the proper range at production and maintained at distribution sites. Women are counselled that salt should be used to replace their household salt, on the approximate daily use (~10 g (three pinches)/day), the proper storage of salt (away from light, heat, humidity, recapping container after use) and use of salt only after cooking/heating of food.

Fortified BEP supplement for malnourished women

In the ENP health centres, women who have mid-upper arm circumferences (MUAC) <23 cm at enrolment, or at any follow-up ANC visit, are provided with a monthly supply of locally produced, micronutrient fortified, corn soya flour blend (Super Cereal, Faffa Food Share Company, Addis Ababa, Ethiopia) at every ANC visit until delivery. The daily corn soya blend supplement (200 g) is provided in addition to normal meals and contains 28 g of protein and 760 kcal (online supplemental web table 2). This protein composition falls within recommendations that a BEP supplement provide~50% of the additional protein requirement in the third trimester (range of 28–36 g for malnourished populations).[34] Micronutrient composition of the provided BEP is also shown in online supplemental web table 2. The fortified BEP supplement meets the Institute of Medicine's recommended levels in pregnancy[35] for vitamins A, D, E, B_2, B_3, B_6, B_{12}, C, calcium and phosphorus (see online supplemental web table 2). Thirty-five sachets (200 g each) are distributed to women at enrolment and at follow-up monthly ANC visits to allow for additional doses in case she is delayed in returning for ANC and/or for potential family sharing practices.

EIMP

Women randomised to the EIMP intervention, receive the following interventions at their first ANC visit >12 weeks (table 2).

Urinary tract infection/asymptomatic bacteriuria

A clean catch midstream urine specimen is collected using a vacutainer with boric acid preservative (Beckton Dickinson). Urine culture and antibiotic susceptibility testing are performed at APHI, the regional laboratory certified by the ENAO (Ethiopian National Accreditation Office)—ISO (International Organization for Standardization) 15189. Antibiotic susceptibility is determined including the Vitek method (bioMerieux, Marcy l'Etoile, France), or Kirby Bauer Disk Diffusion. Urinary tract infections are classified in online supplemental web table 3 and treated with an oral antibiotic-based on antibiotic sensitivity patterns (online supplemental web table 4). Antibiotics are provided to pregnant women at no cost and the first dose is directly observed. Women with severe illness or difficult to treat infections are referred to the

bmjpo: first published as 10.1136/bmjpo-2021-001327 on 13 January 2022. Downloaded from http://bmjpaedsopen.bmj.com/ on April 25, 2024 by guest. Protected by copyright.

Table 2 ENAT Enhanced infection management package (EIMP) components

Infection	EIMP activity
Urinary tract infection/ asymptomatic bacteriuria	*Screen:* Urine culture and antibiotic sensitivity.<br>*Treat:* Initially per clinical protocol, then based on culture results with targeted antibiotic treatment-based on antibiotic resistance patterns.
Sexually transmitted/ reproductive tract infections	*Screen:*<br>1. ***ALL pregnant women*** screened for gonorrhoea and chlamydia using accurate rapid diagnostic nucleic acid amplification testing (Cepheid GeneXpert).<br>2. Pregnant women ***with symptoms*** are additionally screened for trichomonas and bacterial vaginosis (point of care diagnostics, TrichOSM and BVBlue)<br>*Treat:* All positive cases and partners (for gonorrhoea, chlamydia, trichomonas) are treated.<br>*Chlamydia:* azithromycin 1gm po<br>*Gonorrhea:* ceftriaxone 250 gm IM and azithromycin 1gm po<br>*Trichomonas:* metronidazole 2 gm po once<br>*Bacterial vaginosis:* metronidazole 500 mg po bid x 7 days
Geohelminths	Presumptive deworming at enrolment with mebendazole 500 mg or albendazole 400 mg once as per FMOH guideline; followed by stool screen and treatment for parasitic infections at least 4 weeks after initial deworming. If positive, treatment for intestinal parasites per FMOH guidelines.

ENAT, Enhancing Nutrition and Antenatal Infection Treatment; FMOH, Ethiopian Federal Ministry of Health.

Obstetrics Department at Felege Hiwot Hospital. Women provide a test of cure specimens at the following ANC visit.

Sexually transmitted/reproductive tract infections

Women self-collect vaginal specimens (mid-vaginal swab) that are tested for gonorrhoea and chlamydia with the *Xpert CT/NG assay* (*Cepheid*, Sunnyvale, California) using the GeneXpert nucleic acid amplification testing platform at APHI. Chlamydia is treated with azithromycin 1 g orally once and gonorrhoea is treated with ceftriaxone 250 mg intramuscular +azithromycin 1 g orally once. Partner treatment is on a voluntary basis with a regimen as recommended by the Ethiopian sexually transmitted infections management guidelines.[36] A test of cure is obtained at the next ANC visit.

For women who report symptoms of abnormal vaginal discharge, vulvar symptoms or lower abdominal tenderness, additional vaginal swabs are collected for trichomonas and bacterial vaginosis by point of care diagnostics. Trichomonas is tested using the OSOM trichomonas rapid test (Sekisui Diagnostics, Massachusetts). Bacterial vaginosis is tested using Diagnosit BVBLUE test (Gryphus Diagnostics, Knoxville, Tennessee). Trichomonas is treated with metronidazole 2 g orally once, and partners are treated. Bacterial vaginosis is treated with metronidazole 500 mg two times per day for 7 days.

Parasitic intestinal infections

In settings of high geohelminth burden, WHO recommends prophylactic deworming in second and third trimester of pregnancy.[37] At study initiation, mebendazole (500 mg) was provided two times in pregnancy consistent with WHO guidelines. Due to health provider concerns regarding medication package insert information contraindicating use in early pregnancy, in September 2020, the protocol was modified to a single presumptive deworming in the third trimester. In May 2021, with the adoption of new Ethiopian FMOH ANC guidelines allowing earlier provision of antihelminthics in pregnancy, the ENAT protocol was modified to provide presumptive deworming in the second trimester followed by a stool screening and treatment at least 4 weeks later. In the first post-deworming visit, stool is screened for intestinal parasites in the health centre laboratory using wet mount microscopy available at the health centre. Women identified with parasitic infections are treated as per FMOH recommendations (online supplemental web table 4).

Randomisation/allocation

At the first level of randomisation, clusters (ie, health centres) are randomised into one of two nutrition interventions: (a) ENP or (b) standard nutrition care. We performed a constrained randomisation to ensure balance across the two arms of the study for key indicators including: population size, pre-study ANC coverage rates, number of births and travel time to the regional centre of Bahir Dar. We: (1) set reasonable tolerance levels for the restriction variables, (2) created all possible random sequences, where each sequence allocated six health centres to the ENAT Nutrition Package and six health centres to routine care, (3) assessed each sequence as to whether or not it met these restriction criteria and (4) chose randomly from the subset of all such allocation sequences that met the criteria. At the second level of randomisation, we randomised individual pregnant women presenting for ANC at each health centre to receive (a) ENAT EIMP, or (b) standard infection care (figure 2). Each health centre received a pre-generated randomisation list of sequential individual assignments to EIMP or standard care, where assignments were equally allocated to each arm within randomly permuted blocks of size 4, 8 or 12. The randomisation lists were generated separately, by health centre, using a script written by one of the authors (LCM) in R.[38]

bmjpo: first published as 10.1136/bmjpo-2021-001327 on 13 January 2022. Downloaded from http://bmjpaedsopen.bmj.com/ on April 25, 2024 by guest. Protected by copyright.

Table 3 Enhancing Nutrition and Antenatal Infection Treatment study outcomes

**PRIMARY OUTCOMES**	
P1. Newborn weight	Weight of the unclothed infant measured at <72 hours of life.
P2. Newborn length	Infant length measured at <72 hours of life.
**SECONDARY OUTCOMES**	
S1. Gestational age	Gestational age determined by enrolment ultrasound, CRL used if <95 mm (INTERGROWTH 21st), then BPD/FL (WHO Kiserud) used if CRL ≥95 mm or missing.
S2a. Proportion of pregnancies resulting in delivery at <37 weeks gestation	Numerator: number of pregnancies resulting in spontaneous termination of pregnancy at <37 weeks gestation (including preterm live birth or fetal loss (spontaneous pregnancy loss, not due to induced abortion)).<br>Denominator: All pregnancy outcomes.
S2b. Preterm live birth rate	Numerator: Live births <37 weeks of gestation.<br>Denominator: Live births.
S3. Small-for-gestational age (INTERGROWTH)	Numerator: Infants <10% birth weight for GA by sex compared with INTERGROWTH neonatal birthweight standard.[2]<br>Denominator: Live births
S4. Low birth weight	Numerator: Newborns with birth weight (<72 hours of life) <2500 g.<br>Denominator: Live births.<br>We will also assess the outcome of birth weight <2000 g.
S5. Stillbirth rate	Numerator: Stillbirth/fetal death (≥28 weeks gestation) with no signs of life.<br>Denominator: All live births and stillbirths
S6. Newborn head circumference	Head circumference of the infant measured at <72 hours of age.
S7. Newborn weight, length and head circumference for age Z-scores	Infant weight, length and head circumference for age z-scores measured at <72 hours of life, calculated using the INTERGROWTH neonatal standards for size at birth.
S8. Rate of weight gain in pregnancy	Maternal weight gain (kg) per week gestation in the second and third trimester.
S9. Maternal anaemia	Mean haemoglobin concentration in third trimester

BPD, biparietal diameter; CRL, crown rump length; FL, femur length; GA, gestational age.

Outcome measures

The **primary outcomes** are

P1. Newborn weight measured within 72 hours of birth.

P2. Newborn length measured within 72 hours of birth.

The **secondary outcomes** include:

S1. Length of gestation, with gestational age determined by <=24-week pregnancy ultrasonography.

S2a. Proportion of pregnancies resulting in spontaneous delivery at <37 weeks gestation.

S2b. Proportion of livebirths born <37 weeks gestation

S3. Proportion of newborns born small-for-gestational age, as defined by the INTERGROWTH 21st neonatal birth weight standard.

S4. Proportion of newborns born of LBW (<2500 g), as measured within 72 hours of life.

S5. Stillbirth rate.

S6. Newborn head circumference within 72 hours of birth.

S7. Infant Z-scores for weight-for-age, length-for-age, head circumference-for-age within 72 hours of birth.

S8. Maternal gestational weight gain.

S9. Maternal anaemia (third trimester).

The definitions used for each outcome measure is shown in table 3.

Data collection

The timeline of individual participant study visits, measurements and data collection are shown in table 4. All data collection and study measurements are performed by research staff (study nurses or data collectors) after routine ANC visits. Study visits are conducted at the health centre, with the exception of the birth visit that may be conducted at home within 72 hours of delivery, for births occurring at home or outside of the study area. Adherence monitoring visits also occur at the home for those participants who do not return to the health centre for follow-up.

The core of the data collection system is the Survey Solutions platform (World Bank, V.20.08, 2021). Study nurses enter data directly into electronic tablets with programmed validity checks during study visits. Paper forms are used if tablets are temporarily unavailable. The tablets are regularly synchronised to the server on the ACIPH campus. A web-based dashboard supports data collectors, supervisors and investigators in real time management and monitoring of study activities.

Enrolment visit

At the enrolment visit at the health centre, data are collected on the participant's socioeconomic status, basic medical and obstetrical history, pregnancy history, maternal morbidity

bmjpo: first published as 10.1136/bmjpo-2021-001327 on 13 January 2022. Downloaded from http://bmjpaedsopen.bmj.com/ on April 25, 2024 by guest. Protected by copyright.

Table 4 Participant timeline schedule of enrolment, interventions, assessment and visits

	Allocation	Post allocation					
	Prenatal					Postnatal	
Time point	ANC1 [<=24 wks]	ANC2	ANC3 [~3rd tri]	ANC4	ANC5+	Birth	1-month
**Enrolment:**							
Eligibility screen	X						
Informed consent	X						
Allocation	X						
**Interventions:**							
ENP	X	X	X	X	X		
EIMP	X	X	X	X	X		
ENP+EIMP	X	X	X	X	X		
**Assessments:**							
**Mothers**							
Ultrasound	X		X				
Basic medical and obstetrical history	X						
Socioeconomic status	X						
Healthcare costs	X		X				
Food insecurity and dietary intake	X	X	X				X
Maternal stress and depression		X	X				X
Maternal anthropometrics	X	X	X	X	X	X	X
Maternal morbidity	X	X	X	X	X	X	X
Labour and delivery characteristics						X	
**Infants**							
Anthropometrics						X	X
Breastfeeding practices						X	X
Morbidity and mortality						X	X

ANC, antenatal care ; EIMP, enhanced infection management package; ENP, enhanced nutrition package; GA, gestational age.

including COVID-19, food security and dietary intake. A dietary quality questionnaire is administered, which has been used in the Ethiopian context.[39]

A basic abdominal obstetric ultrasound (GE VScan Access) is performed by a trained research nurse at the enrolment visit for pregnancy dating . An intensive ultrasonography training and standardisation was performed by General Electric, Ethiopian Radiography Association and sonographers from Beth Israel Deaconess Hospital (Boston, Massachusetts). Sonographers measure crown-rump length, biparietal diameter (outer to inner), head circumference, femoral length, abdominal circumference in duplicate. Approximately 10% of images are externally reviewed (MS, BJW) for quality control.

Maternal and infant anthropometrics are measured by research staff (nurses, data collectors) at baseline and follow-up visits (table 3). Maternal weight is measured with a digital scale (ADE M317600, Germany; precision 100g) and height is measured using an adult stadiometer (Shorr Productions HeightLite). All measurements are performed two times, with a third measurement done if the difference is greater than the minimal acceptable difference defined by INTERGROWTH 21st.[40] Study nurses and data collectors are trained and standardised in anthropometric measurements at the start of the study and every 6months.

Follow-up ANC visits

During the follow-up ANC visits at the health centre, research staff interview women about their health status, morbidity, pregnancy history/complications, counselling/services received, maternal mental health screen and dietary intake. Data are abstracted from routine ANC records, including blood pressure, laboratory testing results and management. Maternal weight and MUAC are measured. In a subset of women, a semi-quantitative food frequency questionnaire for ~70food items is administered at ANC visits. Haemoglobin is measured at enrolment and third trimester ANC visits (HemoCue 301c). Venous haemoglobin is measured if blood is already being drawn for other purposes, and otherwise capillary haemoglobin is measured.

bmjpo: first published as 10.1136/bmjpo-2021-001327 on 13 January 2022. Downloaded from http://bmjpaedsopen.bmj.com/ on April 25, 2024 by guest. Protected by copyright.

Adherence to each nutritional supplement is assessed at ANC visits. The participant is asked to bring the used IFA bottle, salt container and BEP sachets back to the health centre at each ANC visit. Pill or empty sachet count is done, and the salt container weighed. The participant is also asked to recall the number of sachets and/or pills that were taken in the last 7 days, and since the last visit. For mothers who do not attend scheduled ANC visits, a home visit is made by a data collector to assess adherence and conduct pill/sachet counts and remind the mother to return for ANC and study visits.

A repeat ultrasound is performed in the third trimester to monitor fetal growth and assess the position of the baby. If the fetus is determined to be in non-vertex positioning at the third trimester scan, the nurse recommends that the women deliver in the nearest hospital with caesarean section capacity.

Birth visit

Participants who deliver in health facilities are assessed by research staff based in health centres or hospitals as soon as possible after birth, but within 72 hours of life. Data are gathered from women and from chart review about the delivery and immediate postpartum period. Medical records are reviewed for intrapartum course (eg, vital signs, duration of labour), delivery complications and maternal/neonatal morbidity. For deliveries that occur at home, a home visit is made by research staff as soon as possible on birth notification (within 72 hours). Maternal history is obtained per self-report regarding delivery history/complications, and postpartum maternal/neonatal morbidity.

Infant weight is measured using a high quality, precise digital infant scale (ADE M112600, Germany; precision 5 g). Infant length is measured using a portable infantometer (Perspective Enterprises PE-RILB-LTWT, Michigan USA, precision 1 mm). Recumbent length is recorded to the last completed (not the nearest) millimetre (mm). Head, chest and MUAC is measured to the nearest mm using insertion tapes (Shorr productions, Maryland USA). Daily calibration checks are made before each use of infant weighing scales, and length boards to ensure accuracy of measurement.[41 42]

Postnatal visit

Postnatal visits are made at 4–6 weeks for all participants to collect data on maternal and infant vital status, health, morbidity and anthropometrics. Visits are conducted primarily at the health centre, and home visits are made for those who do not return for follow-up. For infants who follow-up at the health centre for routine postnatal care or immunisations, study visits are additionally conducted at 3 and 6 months.

Cost data

Data regarding the costs of delivery of the ENAT study interventions is collected in all study arms. Costs of interventions include three components: system-level costs, costs incurred by health workers for participating in the interventions and costs incurred by individual patients and families. At the system level, costs are captured using the WHO framework [43] using modified survey tools based on our published survey instruments, cost estimation protocols and procedures that have been validated and used in other LMICs (eg, Rwanda, India);[44–46] Costs incurred by health workers for receiving training include time or money spent for participating in training sessions. For all time spent, monetary value is assigned based on their average hourly wage. Costs incurred by patients include costs or time spent for received care or home visits. For visits in health facilities, we collect self-reported cost data from patients.

Biospecimens

In a subset of consenting women, additional biospecimens will be collected for future analysis. These specimens are shown in online supplemental web table 5.

Statistical analysis

Data collected in this study will enable us to conduct a comprehensive analysis of the impact of interventions randomised at the health centre and individual level. The detailed Statistical Analysis Plan of the ENAT study is published separately at: https://addiscontinentaleduet/. In brief, our statistical approach will have multiple steps. We will describe the health centres and pregnant women enrolled in the study and conduct a descriptive quantitative analysis of variables at multiple levels to assess the degree to which our randomisation scheme resulted in similar subpopulations of pregnant women. We will assess the receipt of and adherence to interventions offered, conduct descriptive analyses of the primary and secondary outcomes, compare the outcomes between intervention groups, assess potential effect modifiers and conduct prespecified subgroup and sensitivity analyses.

Sample size

We have estimated the effect size detectable with 80% power under a cluster-randomised design, with six health centres per study arm. Fixing recruitment of pregnant women to 18 months, we estimated that within this time period the average health centre in the proposed study site would enrol around 200 women into ANC at ≤24 weeks gestation and would yield 112 live born infants weighed within 72 hours of life (assuming~70% of enrolled pregnancies result in a live birth and ~80% are followed-up and weighed within 72 hours). Beyond the above determination of average cluster size, we have additionally made the following assumptions in order to estimate effect sizes detectable with 80% power: (1) mean birth weight and SD as per prior studies in Gondor (mean birth weight of 2900 g, SD 450 g) and (2) variation in distribution of weight between clusters as reflected through an coefficient of variation (k=0.01).[47] In total this includes 2400 pregnant mothers enrolled in 12 health centres, resulting in 1440 live births with a birth weight within 72 hours. This sample size provides 80% power to detect a

bmjpo: first published as 10.1136/bmjpo-2021-001327 on 13 January 2022. Downloaded from http://bmjpaedsopen.bmj.com/ on April 25, 2024 by guest. Protected by copyright.

66 g difference in birth weight between the ENAT EIMP or routine care group in a marginal analysis (ie, irrespective of whether mothers did or did not receive the ENP), and a 90 g difference between ENP versus routine care (marginal analysis).

With the assumptions of clusters and enrolment above, we assumed mean infant length of 49.5 cm (SD 2.4) (based on data from Malawi [48 49] and coefficient of variation k=0.008 (sector level variation in JiVitA study).[47] For the EIMP versus routine care comparison, we would have 80% power to detect a 3.0 mm difference in mean infant length. For the ENP versus non-ENP comparison (marginal analysis) we would have 80% power to detect a 7.8 mm difference in infant length between the women receiving the package of enhanced nutrition-infection compared with standard nutrition care.

Study monitoring

An external Study Monitoring Committee (SMC) is established to monitor the progress of the study, including enrolment, progress indicators and adverse events. The SMC includes an independent Ethiopian obstetrician (Dr Delayehu Bekele) and an epidemiologist (Professor Simon Cousens). The committee met before study initiation, at mid-enrolment and every 6 months to review study progress. Interim analysis will not be performed.

ETHICS AND DISSEMINATION

The study is registered at ISRCTN. Protocol modifications will be communicated to respective Institutional Review Boards and ISRCTN.

Confidentiality

All the data collected for this study is kept strictly confidential at ACIPH on a local encrypted server. Personal identifiers are not used in study-specific forms, aside from the identifier module. Paper copies of data forms for data entry and analysis are stored in a locked file when not in use. Access to data files containing personal identifying information is limited to the principal investigators and key staff.

Ancillary care

ENAT project helps cover the ancillary care related to study participation that is not covered by the health system.

Dissemination plan

ENAT study findings will be disseminated to FMOH, APHI, ARHB, Zonal health departments, woreda health offices, community representatives and to other relevant stakeholders. Involving relevant stakeholders in the dissemination process will help to enhance ownership of the research output and the ultimate integration of findings into programmes. The dissemination will be in the form of presentations in workshops, conferences and symposiums at local, regional, national and international levels as appropriate. In addition, reports and peer-review journal publications will be produced.

DISCUSSION

Despite the high global burden of LBW, preterm birth and fetal growth restriction, few interventions have demonstrated efficacy or effectiveness in the prevention of these adverse birth outcomes. Novel, integrated approaches are needed to make more substantial, scalable and sustainable impacts in order to meet the WHO's Nutrition targets. A critical feature of the ENAT study is its health systems approach and involvement of key local stakeholders in the design of intervention packages and implementation. The packaging/bundling of interventions aims to maximise their potential impact, cost-effectiveness, learning and future scalability. This study will provide important evidence on the role of strengthening ANC programmes to improve maternal health and birth outcomes, and will help inform future ANC policies in Ethiopia. ENAT will contribute to the growing body of knowledge regarding the effectiveness of BEP supplementation during pregnancy. Second, despite the high burden of antenatal infections, there is limited high-quality evidence that interventions to screen and treat prenatal infections improves birth outcomes. To our knowledge, there is even more limited data on the interactions of nutrition and infections in pregnancy and this study will provide novel new insight on the potential for synergistic benefits of an integrated approach. Finally, the study will generate implementation learning on how to optimise delivery of WHO guidelines in resource limited health systems.

Author affiliations
[1]Department of Pediatric Newborn Medicine, Global Advancement of Infants and Mothers, Brigham and Women's Hospital, Boston, Massachusetts, USA
[2]Harvard Medical School, Boston, Massachusetts, USA
[3]Addis Continental Institute of Public Health, Addis Ababa, Ethiopia
[4]Department of International Health, Johns Hopkins Bloomberg School of Public Health, Baltimore, Maryland, USA
[5]Amhara Public Health Institute, Bahir Dar, Ethiopia
[6]Department of Medical Critical Care, Boston Children's Hospital, Boston, Massachusetts, USA
[7]Department of Epidemiology, Harvard TH Chan School of Public Health, Boston, Massachusetts, USA
[8]Departments of Nutrition and Global Health and Population, Harvard TH Chan School of Public Health, Boston, Massachusetts, USA
[9]Division of Global Health Equity, Brigham and Women's Hospital, Boston, Massachusetts, USA
[10]Felege Hiwot Comprehensive Referral Hospital, Bahir Dar, Ethiopia
[11]Department of Obstetrics and Gynecology, Beth Israel Deaconess Medical Center, Boston, Massachusetts, USA
[12]Department of Infectious Disease Epidemiology, London School of Hygiene and Tropical Medicine, London, UK
[13]Debretabor Referral Hospital, Amhara Regional Health Bureau, Bahir Dar, Ethiopia

Twitter Grace J Chan @gracejchan

Acknowledgements We acknowledge all of the ENAT partners for their essential support and contributions to the study, including the Amhara Regional Health Bureau, Amhara Public Health Institute, Ethiopian Federal Ministry of Health, Jhpiego, Ethiopian Society of Obstetrics and Gynaecology, General

bmjpo: first published as 10.1136/bmjpo-2021-001327 on 13 January 2022. Downloaded from http://bmjpaedsopen.bmj.com/ on April 25, 2024 by guest. Protected by copyright.

Electric, and Ethiopian Society of Radiology. We thank and acknowledge Jhpiego and Children's Investment Fund for the support of the ENAT-affilated Project activites that provided foundational comprehensive ANC health systems strengthening efforts in study areas prior to/during the study, including Shoa Girma, Ababa Ayele, Firew Ayalew, and Takele Yeshiwas. We thank APHI and Gizachew Yismaw for support of the laboratory components of the study. We would also like to thank our Study Monitoring Committee, Dr Delayehu Bekele and Professor Simon Cousens.

Contributors Wrote manuscript: ACCL, LCM, FWA, ME and YB. Designed study/protocol: ACCL, FWA, LCM, EB, ME, GJC, PC, SI, CL, RM, AWT, BJW, AW and YB. Designed study materials/study tools: ACCL, FWA, EB, YYB, MMD, ME, NF, IEO, WTK, TS, FS, FT, ST, KY, SI, YK, CL, RM, MDS, FVD, AWT and YB. Trained field data collectors: FWA, EB, YYB, MMD, ME, WTK, TS, FS, FT, ST, KY, AWT, ACCL and YB. Designed data management system: NF, FS, FT, FVD and AW. Trained ultrasonography: MDS, BJW, KY and ST. Critically reviewed/revised manuscript: All authors. All authors have met ICJME criteria.

Funding This work was supported by the Bill & Melinda Gates Foundation grant number OPP1184363.

Disclaimer The funder provided input on the study design, but played no role in the data collection, data analysis, interpretation of data, writing of the report or decision to submit the report for publication.

Map disclaimer The inclusion of any map (including the depiction of any boundaries therein), or of any geographical or locational reference, does not imply the expression of any opinion whatsoever on the part of BMJ concerning the legal status of any country, territory, jurisdiction or area or of its authorities. Any such expression remains solely that of the relevant source and is not endorsed by BMJ. Maps are provided without any warranty of any kind, either express or implied.

Competing interests ACCL reports funding from the Bill & Melinda Gates Foundation, Eunice Kennedy Shriver National Institute of Child Health and Human Development and the WHO. YB reports funding from FORMAS, USAID, National Institute of Environmental Health Sciences and from the Bill & Melinda Gates Foundation. BJW reports funding from National Institute of Environmental Health Sciences and from the Bill & Melinda Gates Foundation.

Patient consent for publication Not applicable.

Ethics approval The Enhancing Nutrition and Antenatal Infection Treatment protocol was approved at Addis Continental Institute of Public Health (001-A1-2019) and Mass General Brigham Institutional Review Board (2018P002479). The Amhara Public Health Institute granted local permission to conduct the study.

Provenance and peer review Not commissioned; externally peer reviewed.

Data availability statement Data are available upon reasonable request. The final data set will be available to the ENAT investigative team and collaborators. Anonymised study data may be made available upon request to the study PIs with approval and in accordance with Ethiopian and US regulatory guidelines.

Supplemental material This content has been supplied by the author(s). It has not been vetted by BMJ Publishing Group Limited (BMJ) and may not have been peer-reviewed. Any opinions or recommendations discussed are solely those of the author(s) and are not endorsed by BMJ. BMJ disclaims all liability and responsibility arising from any reliance placed on the content. Where the content includes any translated material, BMJ does not warrant the accuracy and reliability of the translations (including but not limited to local regulations, clinical guidelines, terminology, drug names and drug dosages), and is not responsible for any error and/or omissions arising from translation and adaptation or otherwise.

Open access This is an open access article distributed in accordance with the Creative Commons Attribution 4.0 Unported (CC BY 4.0) license, which permits others to copy, redistribute, remix, transform and build upon this work for any purpose, provided the original work is properly cited, a link to the licence is given, and indication of whether changes were made. See: https://creativecommons.org/licenses/by/4.0/.

ORCID iDs

Anne CC Lee http://orcid.org/0000-0003-2654-9862
Grace J Chan http://orcid.org/0000-0002-2716-1643

REFERENCES

1 World Health Organization. *Global nutrition targets 2025: low birth weight policy brief*. Geneva, Switzerland, 2014.
2 Blencowe H, Krasevec J, de Onis M, *et al*. National, regional, and worldwide estimates of low birthweight in 2015, with trends from 2000: a systematic analysis. *Lancet Glob Health* 2019;7:e849–60.
3 Katz J, Lee AC, Kozuki N, *et al*. Mortality risk in preterm and small-for-gestational-age infants in low-income and middle-income countries: a pooled country analysis. *Lancet* 2013;382:417–25.
4 Christian P, Lee SE, Donahue Angel M, *et al*. Risk of childhood undernutrition related to small-for-gestational age and preterm birth in low- and middle-income countries. *Int J Epidemiol* 2013;42:1340–55.
5 Blencowe H, Lee ACC, Cousens S, *et al*. Preterm birth-associated neurodevelopmental impairment estimates at regional and global levels for 2010. *Pediatr Res* 2013;74 Suppl 1:17–34.
6 Murray E, Fernandes M, Fazel M, *et al*. Differential effect of intrauterine growth restriction on childhood neurodevelopment: a systematic review. *BJOG* 2015;122:1062–72.
7 Danaei G, Andrews KG, Sudfeld CR, *et al*. Risk factors for childhood stunting in 137 developing countries: a comparative risk assessment analysis at global, regional, and country levels. *PLoS Med* 2016;13:e1002164.
8 Han Z, Mulla S, Beyene J, *et al*. Maternal underweight and the risk of preterm birth and low birth weight: a systematic review and meta-analyses. *Int J Epidemiol* 2011;40:65–101.
9 Kozuki N, Katz J, Lee ACC, *et al*. Short maternal stature increases risk of small-for-gestational-age and preterm births in low- and middle-income countries: individual participant data meta-analysis and population attributable fraction. *J Nutr* 2015;145:2542–50.
10 Peña-Rosas JP, De-Regil LM, Garcia-Casal MN, *et al*. Daily oral iron supplementation during pregnancy. *Cochrane Database Syst Rev* 2015:CD004736.
11 Keats EC, Neufeld LM, Garrett GS, *et al*. Improved micronutrient status and health outcomes in low- and middle-income countries following large-scale fortification: evidence from a systematic review and meta-analysis. *Am J Clin Nutr* 2019;109:1696–708.
12 Imdad A, Bhutta ZA. Maternal nutrition and birth outcomes: effect of balanced protein-energy supplementation. *Paediatr Perinat Epidemiol* 2012;26 Suppl 1:178–90.
13 Ota E, Hori H, Mori R, *et al*. Antenatal dietary education and supplementation to increase energy and protein intake. *Cochrane Database Syst Rev* 2015:CD000032.
14 Buppasiri P, Lumbiganon P, Thinkhamrop J, *et al*. Calcium supplementation (other than for preventing or treating hypertension) for improving pregnancy and infant outcomes. *Cochrane Database Syst Rev* 2011:CD007079.
15 Gilbert NM, O'Brien VP, Hultgren S, *et al*. Urinary tract infection as a preventable cause of pregnancy complications: opportunities, challenges, and a global call to action. *Glob Adv Health Med* 2013;2:59–69.
16 Romero R, Oyarzun E, Mazor M, *et al*. Meta-Analysis of the relationship between asymptomatic bacteriuria and preterm delivery/low birth weight. *Obstet Gynecol* 1989;73:576–82.
17 Honkpéhèdji YJ, Adegbite BR, Zinsou JF, *et al*. Association of low birth weight and polyparasitic infection during pregnancy in Lambaréné, Gabon. *Trop Med Int Health* 2021;26:973–81.
18 Aderoba AK, Iribhogbe OI, Olagbuji BN, *et al*. Prevalence of helminth infestation during pregnancy and its association with maternal anemia and low birth weight. *Int J Gynaecol Obstet* 2015;129:199–202.
19 Abioye AI, McDonald EA, Park S, *et al*. Maternal, placental and cord blood cytokines and the risk of adverse birth outcomes among pregnant women infected with Schistosoma japonicum in the Philippines. *PLoS Negl Trop Dis* 2019;13:e0007371.
20 Goldenberg RL, Hauth JC, Andrews WW. Intrauterine infection and preterm delivery. *N Engl J Med* 2000;342:1500–7.
21 Christensen DL, Kapur A, Bygbjerg IC. Physiological adaption to maternal malaria and other adverse exposure: low birth weight, functional capacity, and possible metabolic disease in adult life. *Int J Gynaecol Obstet* 2011;115 Suppl 1:S16–19.
22 Krawinkel MB. Interaction of nutrition and infections globally: an overview. *Ann Nutr Metab* 2012;61 Suppl 1:39–45.
23 Vohr BR, Poggi Davis E, Wanke CA, *et al*. Neurodevelopment: the impact of nutrition and inflammation during preconception and pregnancy in low-resource settings. *Pediatrics* 2017;139:S38–49.
24 Kim H, Hwang J-Y, Ha E-H, *et al*. Association of maternal folate nutrition and serum C-reactive protein concentrations with gestational age at delivery. *Eur J Clin Nutr* 2011;65:350–6.
25 Kutlesic V, Brewinski Isaacs M, Freund LS, *et al*. Executive summary: research gaps at the intersection of pediatric neurodevelopment, nutrition, and inflammation in low-resource settings. *Pediatrics* 2017;139:S1–11.

bmjpo: first published as 10.1136/bmjpo-2021-001327 on 13 January 2022. Downloaded from http://bmjpaedsopen.bmj.com/ on April 25, 2024 by guest. Protected by copyright.

26 Collin SM, Baggaley RF, Pittrof R, *et al*. Could a simple antenatal package combining micronutritional supplementation with presumptive treatment of infection prevent maternal deaths in sub-Saharan Africa? *BMC Pregnancy Childbirth* 2007;7:6.
27 World Health Organization (WHO). *Who recommendations on Antental care for a positive pregnancy experience*, 2016.
28 Central Statistical Agency CSAE, Icf. *Ethiopia demographic and health survey 2016*. Addis Ababa, Ethiopia: CSA and ICF, 2017.
29 Ethiopia Mini. *Demographic and health survey 2019: key indicators*. Rockville, Maryland, USA: EPHI and ICF, Ethiopian Public Health Institute (EPHI) [Ethiopia] and ICF, 2019.
30 Shiferaw MB, Zegeye AM, Mengistu AD. Helminth infections and practice of prevention and control measures among pregnant women attending antenatal care at Anbesame health center, Northwest Ethiopia. *BMC Res Notes* 2017;10.
31 Belyhun Y, Medhin G, Amberbir A, *et al*. Prevalence and risk factors for soil-transmitted helminth infection in mothers and their infants in Butajira, Ethiopia: a population based study. *BMC Public Health* 2010;10:21.
32 Workneh F, Eglovitch M, Shiferaw T, *et al*. Dietary practices among pregnant women in rural Amhara, Ethiopia. *Curr Dev Nutr* 2021;5:698.
33 Baye E, Abate FW, Eglovitch M, *et al*. Effect of birthweight measurement quality improvement on low birthweight prevalence in rural Ethiopia. *Popul Health Metr* 2021;19:35.
34 Members Of An Expert Consultation On Nutritious Food Supplements For Pregnant And Lactating Women. Framework and specifications for the nutritional composition of a food supplement for pregnant and lactating women (PLW) in undernourished and low income settings. *Gates Open Res* 2019.
35 Institute of Medicine. *Dietary reference intakes (DRIs): recommended dietary allowances and adequate intakes*. Washington, DC: The National Academies Press.
36 Federal Democratic Republic of Ethiopia Ministry of Health. *National guidelines for the management of sexually transmitted infections using syndromic*. Addis Ababa, Ethiopia, 2015.
37 World Health Organization. Soil-transmitted helminth infections, 2020. Available: https://www.who.int/news-room/fact-sheets/detail/soil-transmitted-helminth-infections [Accessed 8 Sep 2021].
38 R Core Team. *R: a language and environment for statistical computing*. Vienna, Austria: R Foundation for Statistical Computing, 2021.
39 Workicho A, Belachew T, Feyissa GT, *et al*. Household dietary diversity and animal source food consumption in Ethiopia: evidence from the 2011 welfare monitoring survey. *BMC Public Health* 2016;16:1192.
40 International Fetal and Newborn Growth Consortium. *The International Fetal and Newborn Growth Standards for the 21st Century(INTERGROWTH-21st) Study Protocol*, 2009.
41 de Onis M, Onyango AW, Borghi E, *et al*. Comparison of the world Health organization (who) child growth standards and the National center for health Statistics/WHO international growth reference: implications for child health programmes. *Public Health Nutr* 2006;9:942–7.
42 Bloem M. The 2006 who child growth standards. *BMJ* 2007;334:705–6.
43 World Health Organization. *The world health report 2000 - Health systems: improving performance*. Geneva, Switzerland: World Health Organization, 2000.
44 Lu C, Tsai S, Ruhumuriza J, *et al*. Tracking rural health facility financial data in resource-limited settings: a case study from Rwanda. *PLoS Med* 2014;11:e1001763.
45 Tsai S, Umugiraneza G, Ruhumuriza J. *Training manual on tracking financial data in health facilities in Kayonza and Kirehe*. Boston, MA: Program in Global Health Economics and Social Change, Harvard Medical School, 2013.
46 Lu C, Tsai S, Ruhumuriza J. *Obtaining financing data for local health facilities in two districts of Rwanda: general guidelines*. Boston, MA: Program in Global Helath Economics and Social Change, Harvard Medical School, 2013.
47 West KP, Shamim AA, Mehra S, *et al*. Effect of maternal multiple micronutrient vs iron-folic acid supplementation on infant mortality and adverse birth outcomes in rural Bangladesh: the JiVitA-3 randomized trial. *JAMA* 2014;312:2649–58.
48 Luntamo M, Kulmala T, Mbewe B, *et al*. Effect of repeated treatment of pregnant women with sulfadoxine-pyrimethamine and azithromycin on preterm delivery in Malawi: a randomized controlled trial. *Am J Trop Med Hyg* 2010;83:1212–20.
49 Luntamo M, Kulmala T, Cheung YB, *et al*. The effect of antenatal monthly sulphadoxine-pyrimethamine, alone or with azithromycin, on foetal and neonatal growth faltering in Malawi: a randomised controlled trial. *Trop Med Int Health* 2013;18:386–97.

bmjpo: first published as 10.1136/bmjpo-2021-001327 on 13 January 2022. Downloaded from http://bmjpaedsopen.bmj.com/ on April 25, 2024 by guest. Protected by copyright.
