# Supplementary material for: The impact of enhancing nutrition and antenatal infection treatment on birth outcomes in Amhara, Ethiopia: a pragmatic factorial, cluster-randomised clinical effectiveness study
Source: BMJ Glob Health. 2025 Jun 18;10(6):e016264. doi: 10.1136/bmjgh-2024-016264 (PMC12181995; doi:10.1136/bmjgh-2024-016264)
Supplement: online supplemental file 2 [file bmjgh-10-6-s002.docx]

**Supplementary material for:**

**The Impact of Enhancing nutrition and Antenatal infection Treatment on birth outcomes in Amhara, Ethiopia: a pragmatic randomized clinical effectiveness study**

**Appendix Table of Contents**

[Figure S1. ENAT Study Site Map with Health Center Allocation from Protocol Paper 1](#_Toc163683152)

[Table S1. Balanced Energy Protein Supplement Composition (FAFFA Super Cereal) 2](#_Toc163683153)

[Table S2. Classification of Urinary Tract Infections (UTI) in ENAT study 3](#_Toc163683154)

[Figure S2. Classification and Initial Management of Urine Culture results in the ENAT EIMP arm. 4](#_Toc163683155)

[Figure S3. Antibiotic Management of UTI in ENAT EIMP arm 5](#_Toc163683156)

[Figure S4. Management of Sexual/Reproductive Tract Infections in ENAT EIMP Arm. 6](#_Toc163683157)

[Table S3. Recommended Treatments for Parasitic Stool Infections 7](#_Toc163683158)

[Table S4. ENAT Study Outcomes Definitions 8](#_Toc163683159)

[Table S5. ENAT Study Visits and Data collection 9](#_Toc163683160)

[Table S6. ENAT Gestational Age Algorithm 10](#_Toc163683161)

[Table S7. ENAT Cluster (Health Center) Level Baseline Randomization Balance 11](#_Toc163683162)

[Table S8. Characteristics of Women-Infants by Status of Outcome Availability 12](#_Toc163683163)

[Table S9. ENAT Cluster-Specific Primary Outcome – Newborn Weight Measured at <72 hrs 14](#_Toc163683164)

[Table S10. ENAT Cluster-Specific Co-Primary Outcome – Newborn Length Measured at <72 hrs 15](#_Toc163683165)

[Table S11. Effects of Enhanced Nutrition Package on Pregnancy Outcomes, Including Imputed Missing Outcome Data 16](#_Toc163683166)

[Table S12. Effects of Enhanced Infection Management Package on Pregnancy Outcomes, Results Including Imputed Missing Outcome Data 17](#_Toc163683167)

[Table S13. Effects of ENP+EIMP Package on Pregnancy Outcomes, Results Including Imputed Missing Outcome Data 18](#_Toc163683168)

[Table S14. Effects of Enhanced Nutrition Package on Birth size: Subgroup analysis MUAC <23 cm 19](#_Toc163683169)

[Table S15. A priori Sub-group Analysis of Primary Outcomes (ITT) 20](#_Toc163683170)

Table S16. Maternal Morbidity by Study Arm 21

# Figure S1. ENAT Study Site Map with Health Center Allocation from Protocol Paper

**
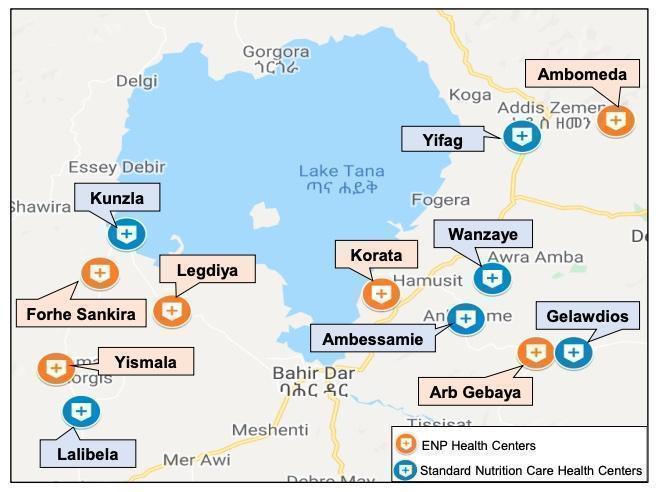
**

| **ENP Health Center** | **Standard Nutrition Health Center** |
| --- | --- |
| Ambomeda | Ambessamie |
| Arb Gebaya | Gelawdios |
| Forhe Sankira | Kunzla |
| Korata | Lalibela |
| Legdiya | Wanzaye |
| Yismala | Yifag |

# Table S1. Balanced Energy Protein Supplement Composition (FAFFA Super Cereal)

Dose: 200 g sachet once daily to supplement regular meals provided for women with MUAC <23 cm

| **Nutrient** | **Content (per 200g)** | **Unit** | **Recommended Ranges in Pregnancy** | |
| --- | --- | --- | --- | --- |
| **Macronutrient** |  |  | **BMGF Expert Consultation^Δ^** | |
| Energy (Kcal) | 760 | kcal | 250-500 kcal  in high-risk population portion size can be doubled | |
| Protein | 28 | g | 16 g (50% of additional protein requirement in 3^rd^ trimester) | |
| Fat | 12 | % | 10-60% of energy | |
| **Micronutrient** |  |  | **IOM**  **Minimum/Target (EAR)*** | **IOM**  **Maximum (RDA)*** |
| Vitamin A | 2076 | mcg RE | 550 | 770 |
| Vitamin D3 | 22.08 | µg | 10 | 15 |
| Vitamin E TE | 16.6 | mg | 16 | 19 |
| Vitamin K1 | 60 | µg | 72 | 90 |
| Vitamin B1 | 0.4 | mg | 1.2 | 1.4 |
| Vitamin B2 | 2.8 | mg | 1.3 | 1.6 |
| Niacin B3 | 16 | mg | 14 | 18 |
| Vitamin B6 | 2 | mg | 1.7 | 2 |
| Folic Acid, B9 | 220 | µg | 400 | 600 |
| Vitamin B12 | 4 | µg | 2.4 | 2.8 |
| Vitamin C | 180 | mg | 100 | 120 |
| Iron | 8 | mg | 22 | 27 |
| Zinc | 10 | mg | 15 | 20 |
| Iodine | 80 | µg | 209 | 290 |
| Copper | 0 | mg | 1.0 | 1.3 |
| Selenium | 0 | µg | 60 | 70 |
| Calcium | 724 | mg | 500 | 1000 |
| Phosphorus | 560 | mg | 300 | 700 |

BMFG= Bill & Melinda Gates Foundation. EAR=estimated average requirement. IOM= Institutes of Medicine. MUAC=mid-upper arm circumference. RDA= recommended dietary allowance.

Δ Bill and Melinda Gates Foundation Expert Consultation on Framework and Specifications for Nutritional Composition of a Food Supplement for Pregnant and Lactating Women in Undernourished and Low-Income Settings. April 2017

*IOM. Dietary Reference Intakes: The Essential Guide to Nutrient Requirements. The National Academies Press, Washington, D.C., 2006. EAR was used for minimum value and RDA for maximum value.

# Table S2. Classification of Urinary Tract Infections (UTI) in ENAT study

| **UTI Terminology** | **Definition** |
| --- | --- |
| High-burden growth | bacteriuria of >10^5^ colony forming units (CFU) per 1mL of urine of a single uropathogen |
| Intermediate growth | bacteriuria with >10^3^ -10^5^ CFU/mL of a single uropathogen, |
| Contamination | bacterial growth of >3 micro-organism OR growth of a non-urinary tract pathogen. |
| UTI symptoms | dysuria, urinary frequency, urinary urgency, hematuria, abdominal pain, fever, OR flank pain |
| Symptomatic intermediate growth | women with intermediate burden growth and UTI symptoms (as above) |
| Asymptomatic bacteriuria | women with high burden bacterial growth without UTI symptoms |
| Cystitis | women with positive urine culture (high burden or intermediate growth) and symptoms of dysuria, urinary frequency, hematuria, urinary urgency or suprapubic tenderness, without upper urinary tract symptoms (fever, chills, flank or back pain) |
| Pyelonephritis | women with positive urine culture and systemic symptoms (fever, chills, flank pain or back pain) |

# Figure S2. Classification and Initial Management of Urine Culture results in the ENAT EIMP arm.

ANC= antenatal care. APHI=Amhara Public Health Institute. CFU= colony-forming units. EIMP= Enhanced infection management package. UTI= urinary tract infection.


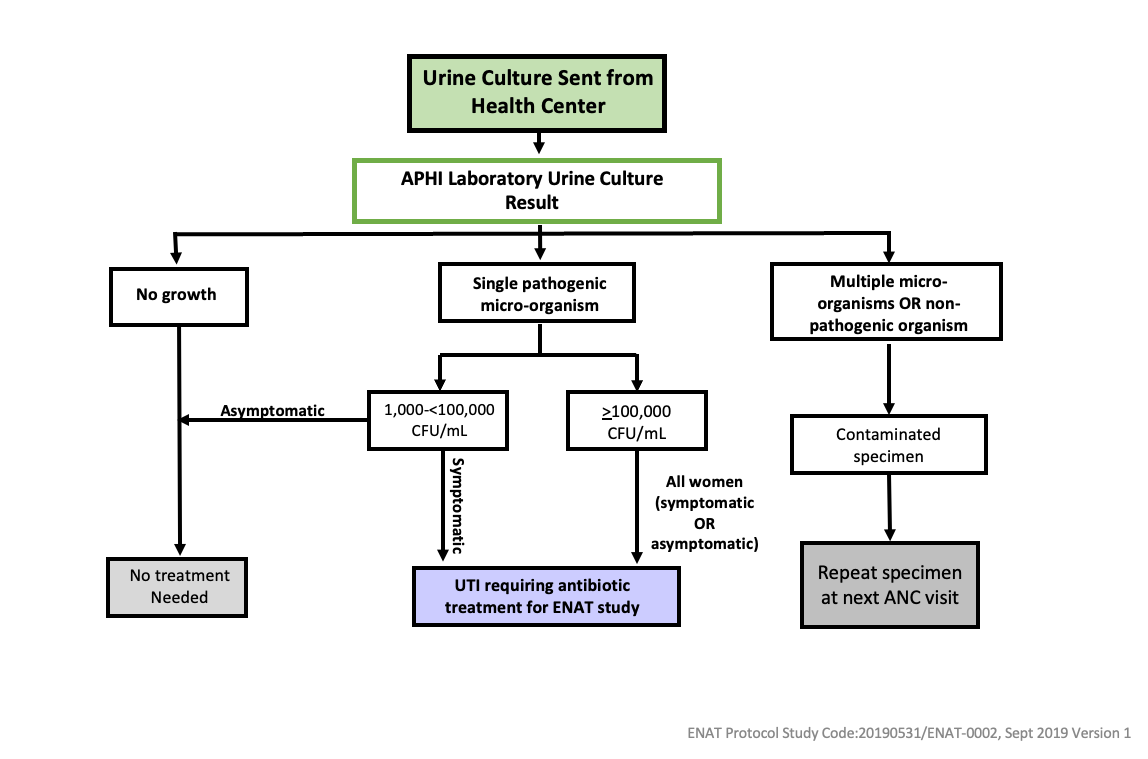


# Figure S3. Antibiotic Management of UTI in ENAT EIMP arm


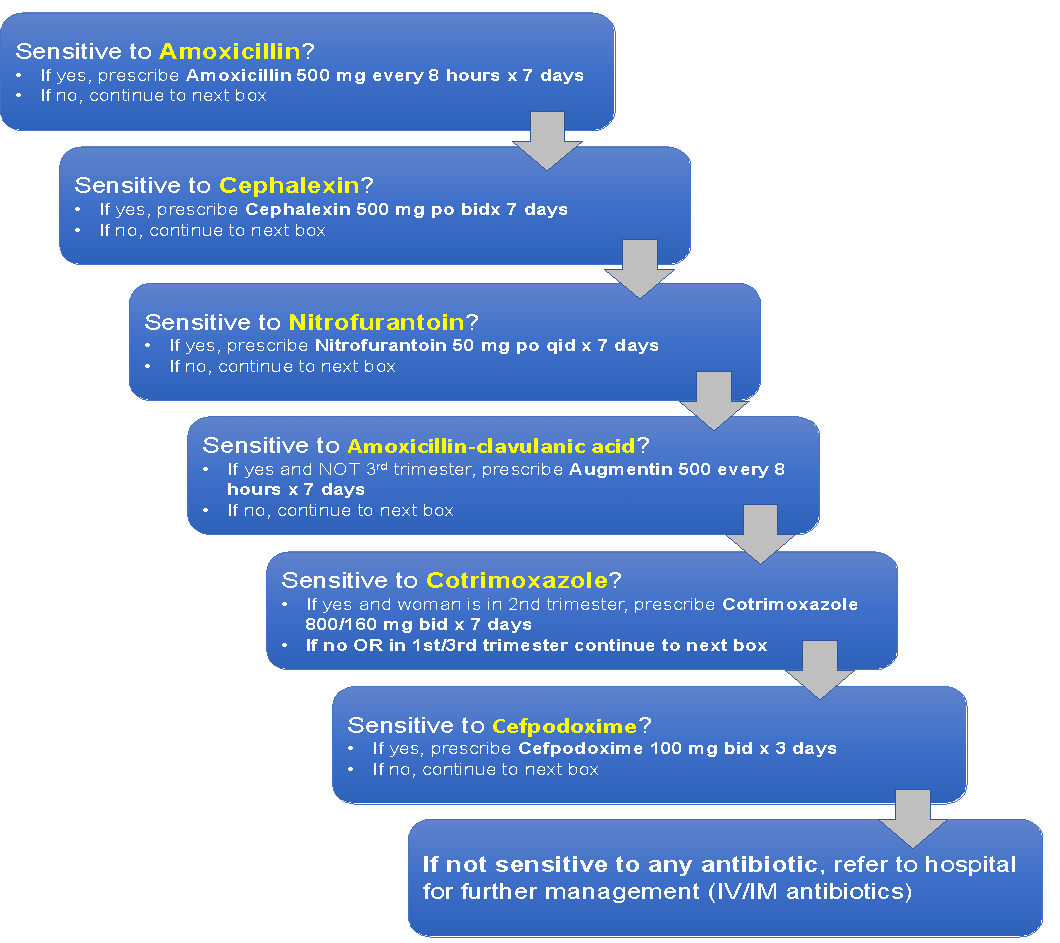


# Figure S4. Management of Sexual/Reproductive Tract Infections in ENAT EIMP Arm.

ANC= antenatal care. APHI=Amhara Public Health Institute. BV= bacterial vaginosis. CT/NG= Chlamydia trachomatis/Neisseria gonorrhoeae. EIMP= Enhanced infection management package. FMOH= Federal Ministry of Health. POC= point of care.


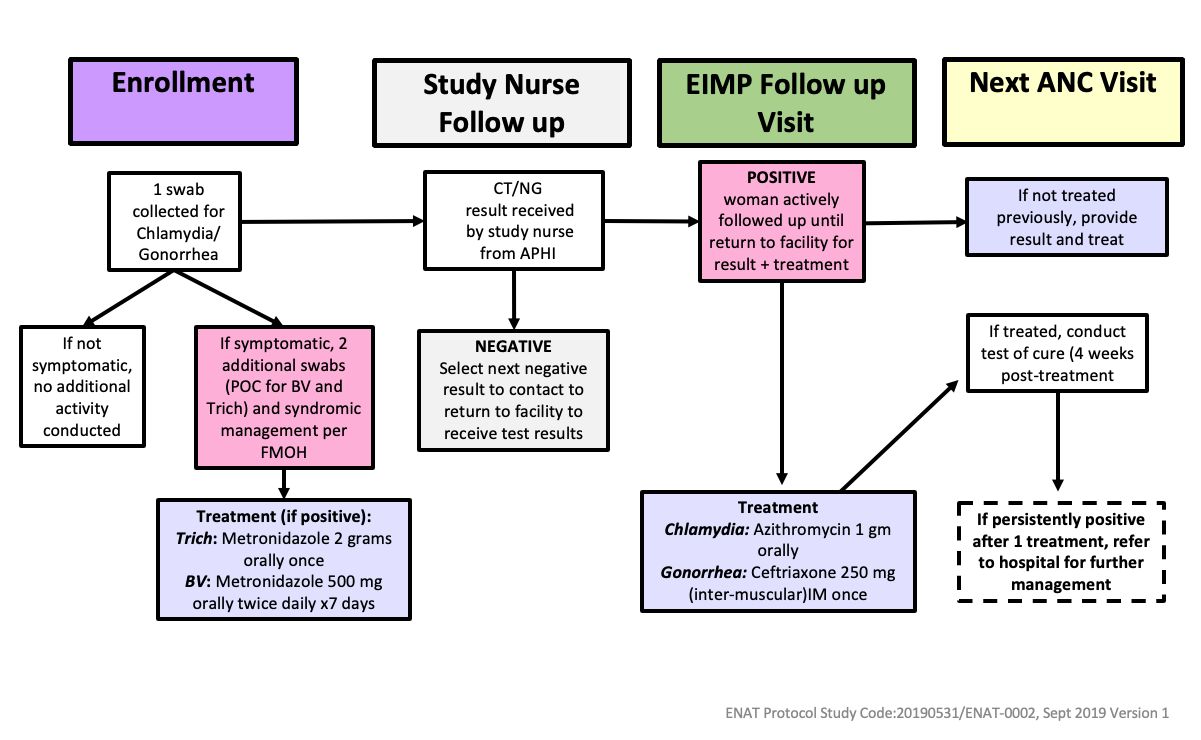


# Table S3. Recommended Treatments for Parasitic Stool Infections

**FMHACA Standard Treatment Guideline, 3^rd^ Edition 2014**

| **Intestinal parasite** | **Recommended treatment, and alternative** |
| --- | --- |
| *Entameoba Histolytica* | Metronidazole 500 mg P.O. TID x 5-7 days |
| *Giardia lamblia* | Tinidazole 2 gm po single dose  Or Alternative:  Metronidazole 500 mg P.O. TID x 5 days |
| Ascariasis  *Ascaris lumbricoids* | Mebendazole, 500mg P.O. once or  Albendazole, 400mg P.O. as a single dose  Or Alternative:  Pyrantel pamoate, 700mg P.O. as a single dose |
| Hookworm infestation  *Necator americanus or Ancylostoma duodenale* | Mebendazole, 500mg stat or 100mg P.O. BID for 3 days  Albendazole, 400mg P.O. as a single dose  Or Alternative:  Pyrantel pamoate, 700mg P.O. as a single dose |
| Enterobiasis  *Enterobius Vermicularis* | Mebendazole, 100mg P.O. BID for 3 days or  Or Alternative:  Albendazole, 400mg P.O. as a single dose |
| Trichuriasis  *T.tricura [Whipworm]* | Mebendazole, 500mg P.O. single dose or,  Or Alternative:  Albendazole, 400mg, P.O. for three days |
| Taeniasis    *T.saginata or T.solium* | Praziquantel P.O. 600mg or 10mg/Kg, single dose  Or Alternative:  Niclosamide, 2g in a single dose P.O. |
| *Hymenolepis nana* | Praziquantel, 25mg/kg or 1800mg P.O. single dose  Alternative:  Niclosamide, 2g P.O. on the first day followed by 1g QD for 6 days |
| *Schistosoma Mansoni* | Praziquantel P.O. 1200 mg single dose (or 600 mg po in 2 doses) |
| Strongyloidiasis  *Strongloidexs stercolaries* | Albendazole 400mg P.O. BID for three consecutive days. |

​​BID= twice per day. FMHACA= Food, Medicine and Health Care Administration and Authority (of Ethiopia). P.O.= per os (orally). QD= once per day. TID= three times per day.

# Table S4. ENAT Study Outcomes Definitions

| **Primary outcomes** | |
| --- | --- |
| **P1. Newborn weight** | Weight of the unclothed infant measured at <72 hours of life |
| **P2. Newborn length** | Infant length measured at <72 hours of life |
| **Secondary outcomes** | |
| **S1. Gestational age** | Gestational age determined by enrollment ultrasound, CRL used if <95 mm (INTERGROWTH-21st), then BPD/FL (WHO Kiserud) used if CRL>95 mm or missing |
| **S2a. Proportion of pregnancies resulting in preterm delivery** | Numerator: number of pregnancies resulting in spontaneous termination of pregnancy from 24 to <37 weeks [including preterm live birth or fetal loss (spontaneous pregnancy loss 24 to <37 weeks, not due to induced abortion)].  Denominator: All pregnancy outcomes >24 weeks |
| **S2b. Preterm live birth** | Numerator: Live births <37 weeks of gestation  Denominator: All livebirths |
| **S3. Small for gestational age** | Numerator: <10% birth weight for GA by sex compared to INTERGROWTH-21^st^ reference^a^  Denominator: All livebirths |
| **S4. Low birthweight** | Numerator: Low birthweight is defined as birthweight (measured within the first 72 h of life) of <2500 g  Denominator: All livebirths |
| **S5. Stillbirth** | Numerator: Stillbirth (≥28 weeks gestation)- fetal death with no signs of life  *A preterm stillbirth is defined as an infant born without signs of life (no spontaneous crying, breathing, and/or movement) at 28 to <37 weeks gestation.*   - *A term stillbirth is defined as an infant born without signs of life at >37 wks*   Denominator: All live births and stillbirths >28 weeks |
| **S6. Newborn head circumference** | Head circumference of the infant measured at <72 hours of age |
| **S7. Newborn weight-, length-, and head circumference-for-age Z-scores** | Infant weight, length, and head circumference for age z-scores measured at <72 hours of life, calculated using the INTERGROWTH-21^st^ reference^a^ for size at birth. |

BPD=biparietal diameter. CRL=crown rump length. FL=femur length. GA= gestational age. WHO= World Health Organization.

^a^ Villar J, et al. International standards for newborn weight, length, and head circumference by gestational age and sex: the Newborn Cross-Sectional Study of the INTERGROWTH-21st Project. Lancet. 2014;384(9946):857-868.

# Table S5. ENAT Study Visits and Data collection

|  | **STUDY PERIOD** | | | | | | |
| --- | --- | --- | --- | --- | --- | --- | --- |
|  |  | **Post-allocation** | | | | | |
|  | **Allocation** | **Prenatal** | | | | **Postnatal** | |
| **TIMEPOINT** | **Enrolment**  **<24 weeks** | ***ANC1*** | ***ANC2*** | ***ANC3*** | ***etc.*** | ***Birth*** | ***1 month*** |
| **ENROLMENT:** | | | | | | | |
| **Eligibility screen** | X |  |  |  |  |  |  |
| **Informed consent** | X |  |  |  |  |  |  |
| **Allocation** | X |  |  |  |  |  |  |
| **INTERVENTIONS:** | | | | | | | |
| **ENP** | X | X | X | X | X |  |  |
| **EIMP** | X | X | X | X | X |  |  |
| **ENP + EIMP** | X | X | X | X | X |  |  |
| **ASSESSMENTS:** | | | | | | | |
| ***MOTHERS*** |  | | | | | | |
| ***US (fetal growth & GA determination)*** | X |  |  | X |  |  |  |
| ***Basic medical & obstetric history*** | X |  |  |  |  |  |  |
| ***Socioeconomic status*** | X |  |  |  |  |  |  |
| ***Health care costs*** | X |  | X |  |  |  |  |
| ***Food insecurity and Dietary Intake*** | X | X |  | X |  |  |  |
| ***Maternal stress and depression*** |  | X |  | X |  |  | X |
| ***Maternal anthropometrics*** | X | X | X | X | etc. | X | X |
| ***Maternal morbidity*** | X | X | X | X | etc. | X | X |
| ***Labor and Delivery characteristics*** |  |  |  |  |  | X |  |
| ***Assessment of home environment*** |  |  |  |  |  | X |  |
| ***INFANTS*** |  | | | | | | |
| ***Anthropometrics*** |  |  |  |  |  | X | X |
| ***Breast feeding practices*** |  |  |  |  |  | X | X |
| ***Morbidity and mortality*** |  |  |  |  |  | X | X |

ANC= antenatal care. EIMP= enhanced infection management package. ENP= enhanced nutrition package. GA= gestational age. US= ultrasound.

# Table S6. ENAT Gestational Age Algorithm

**Hierarchy of Regression equations for predicting GA from fetal measurements.**

| **Hierarchy** (in order of preference, descending priority) | **Fetal measurements** | **Regression equations** | **Citation** |
| --- | --- | --- | --- |
| ≤13 6/7 wks  (ie. if CRL <95mm) | 1. CRL (mm) | GA1_CRL= 40.9041 + (3.21585 × CRL0.5 ) + (0.348956 × CRL) | Intergrowth-21^a^ |
| 14 0/7 to 42 0/7 wks | 2. HC (mm), FL (mm) | GA2_HCFL (wk) =(exp(0.03243*(ln(HC))2 +0.001644*FL*ln(HC) +3.813))/7; SD=0.04009*GA-1.149;, FL and HC in mm | Intergrowth-21^b^ |
|  | 3. HC (mm) | GA3_HC (wk) = (exp(0.05970*(ln(HC)2) +0.000000006409*HC3 +3.3258))/7; SD=0.6492*(GA*0.01)3 +2.991; HC in mm | Intergrowth-21^b^ |
|  | 4. BPD, FL (cm) | GA4_BPDFL (wk) = 10.50 + 0.197(BPD)(FL) + 0.9500(FL) + 0.7300(BPD) | Hadlock |
|  | 5. BPD (cm) | GA5_BPDHad (wk) = 9.54 + 1.482(BPD) + 0.1676(BPD)2 | Hadlock |
|  | 6. FL (cm) | GA6_FLHad (wk) = 10.35 + 2.460(FL) + 0.170(FL)2 | Hadlock |

BPD= biparietal diameter. CRL= crown-rump length. FL= femur length. GA= gestational age. HC= head circumference.

^a^ Papageorghiou AT, Kennedy SH, Salomon LJ, et al. International standards for early fetal size and pregnancy dating based on ultrasound measurement of crown-rump length in the first trimester of pregnancy. *Ultrasound Obstet Gynecol*. 2014;44(6):641-648.

^b^ Papageorghiou AT, Kemp B, Stones W, et al. Ultrasound-based gestational-age estimation in late pregnancy. *Ultrasound Obstet Gynecol*. 2016;48(6):719-726.

# Table S7. ENAT Cluster (Health Center) Level Baseline Randomization Balance

|  | **Nutrition**  **(clusters = 6)** | | **Routine nutrition care**  **(clusters = 6)** | |
| --- | --- | --- | --- | --- |
|  | **Mean** | **SD** | **Mean** | **SD** |
| Average Distance to Bahir Dar (miles) | 37.1 | 5 | 42 | 8.5 |
| Average population served | 36209 | 11322 | 31720 | 12512 |
| Annual ANC visits/health center | 880 | 248 | 789 | 405 |
| Annual births/health center | 337 | 103 | 357 | 240 |
| % facility delivery | 87% | 12% | 77% | 26% |
| Average cluster size  (enrolled women per health center) | 212 | 53 | 217 | 53 |

# Table S8. Characteristics of Women-Infants by Status of Outcome Availability

| **Characteristics of Women-Infants for those with Missing Newborn Weight Measurement** | | | | | | | | |
| --- | --- | --- | --- | --- | --- | --- | --- | --- |
|  | **ENP arm** | | |  | **Routine Nutrition Care (Not-ENP) arm** | | |  |
|  | Weight measured <72 hours | Weight measured >72 hours | No weight available | p value | Weight measured <72 hours | Weight measured >72 hours | No weight available | p value |
| Livebirths, n | 861 | 184 | 44 |  | 749 | 189 | 87 |  |
| Maternal age, mean (SD), yrs | 26.2 (5.6) | 25.6 (5.6) | 24.6 (4.7) | 0.060 | 26.1 (5.3) | 27.3 (5.4) | 26.5 (5.8) | 0.023 |
| Multiparous, n (%) | 616 (71.5) | 125 (67.9) | 21 (47.7) | 0.003 | 523 (69.8) | 134 (72.0) | 60 (69.0) | 0.813 |
| Maternal BMI <18.5 kg/m^2^, n (%) | 168 (19.5) | 32 (17.4) | 9 (20.5) | 0.840 | 102 (13.7) | 14 (7.5) | 18 (20.7) | 0.013 |
| Maternal MUAC <23 cm, n (%) | 258 (29.9) | 42 (22.8) | 14 (31.8) | 0.138 | 179 (25.9) | 49 (29.0) | 31 (38.3) | 0.057 |
| Education (primary or low), % | 79.1% | 82.6% | 59.0% | 0.001 | 70.8% | 80.0% | 80.4% | 0.022 |
| Occupation, n (%) |  |  |  | 0.004 |  |  |  | 0.004 |
| Informal | 227 (26.5) | 36 (19.6) | 9 (20.4) |  | 266 (35.6) | 61 (32.8) | 20 (23.0) |  |
| Agriculture/daily laborer | 472 (55.1) | 119 (64.7) | 19 (43.2) |  | 297 (39.6) | 92 (49.5) | 50 (57.5) |  |
| Waged occupation | 158 (18.4) | 29 (15.8) | 16 (36.4) |  | 185 (24.7) | 33 (17.7) | 17 (19.5) |  |
| Distance from Health Center, mean (SD), mi | 35.4 (28.5) | 41.7 (30.9) | 41.7 (33.8) | 0.016 | 38.9 (32.5) | 46.7 (33.7) | 50.0 (35.7) | 0.001 |
| Place of Birth, n (%) |  |  |  | <0.001 |  |  |  | <0.001 |
| Hospital | 143 (16.6) | 87 (47.3) | 12 (27.3) |  | 116 (15.5) | 55 (29.1) | 22 (27.5) |  |
| Health center | 597 (69.3) | 36 (19.6) | 16 (36.4) |  | 528 (70.5) | 45 (23.8) | 26 (32.5) |  |
| Home/others | 121 (14.1) | 61 (33.1) | 16 (36.4) |  | 105 (14.0) | 89 (47.1) | 32 (40.0) |  |
| Gestational age of pregnancy at outcome, mean (SD), wks | 39.9 (2.0) | 39.7 (2.2) | 38.3 (3.9) | <0.001 | 39.8 (2.1) | 39.1 (3.4) | 39.1 (3.4) | 0.044 |
| Preterm birth, n (%) | 55 (6.4) | 22 (12.0) | 7 (17.5) | <0.001 | 63 (8.4) | 13 (6.9) | 14 (17.7) | 0.012 |

BMI= body mass index. BW= birth weight. ENP= enhanced nutrition package. MUAC= mid-upper arm circumference.

| **Characteristics of Women-Infants for those with Missing Newborn Length Measurement** | | | | | | | | |
| --- | --- | --- | --- | --- | --- | --- | --- | --- |
|  | **ENP arm** | | |  | **Routine Nutrition Care (Not-ENP) arm** | | |  |
|  | Length measured <72 hours | Length measured >72 hours | No length available | p value | Length measured <72 hours | Length measured >72 hours | No length available | p value |
| Livebirths, n | 839 | 182 | 68 |  | 709 | 182 | 132 |  |
| Maternal age, mean (SD), yrs | 26.3 (5.6) | 25.6 (5.6) | 24.1 (4.3) | 0.003 | 26.2 (5.3) | 27.3 (5.4) | 26.0 (5.3) | 0.021 |
| Multiparous, n (%) | 606 (72.2) | 124 (68.1) | 32 (47.1) | <0.001 | 501 (70.7) | 132 (73.7) | 83 (62.9) | 0.104 |
| Maternal BMI <18.5, n (%) | 164 (19.6) | 32 (17.6) | 13 (19.1) | 0.980 | 98 (13.9) | 14 (7.8) | 22 (16.7) | 0.063 |
| Maternal MUAC <23, n (%) | 253 (30.1) | 42 (23.1) | 19 (27.9) | 0.159 | 174 (26.7) | 46 (28.0) | 39 (31.7) | 0.521 |
| Education (primary or low), % | 79.5% | 82.4% | 62.3% | 0.030 | 71.4% | 80.0% | 73.5% | 0.033 |
| Occupation, n (%) |  |  |  | 0.008 |  |  |  | 0.047 |
| Informal | 218 (26.1) | 36 (19.8) | 18 (26.5) |  | 251 (35.4) | 57 (31.8) | 37 (28.0) |  |
| Agriculture/daily laborer | 463 (55.5) | 118 (64.8) | 29 (42.6) |  | 285 (40.3) | 91 (50.8) | 63 (47.7) |  |
| Waged occupation | 154 (18.4) | 28 (25.4) | 21 (30.9) |  | 172 (24.3) | 31 (17.3) | 32 (24.2) |  |
| Distance from Health Center, mean (SD), miles | 35.7 (28.6) | 42.0 (31.0) | 35.9 (31.6) | 0.030 | 39.4 (32.8) | 46.8 (33.9) | 43.9 (34.1) | 0.018 |
| Place of birth, n (%) |  |  |  |  |  |  |  |  |
| Hospital | 130 (15.5) | 86 (47.3) | 26 (38.2) | <0.001 | 95 (13.4) | 55 (30.2) | 42 (33.6) | <0.001 |
| Health center | 588 (70.1) | 35 (19.2) | 26 (38.2) |  | 509 (71.8) | 41 (22.5) | 48 (38.4) |  |
| Home/others | 121 (14.4) | 61 (33.5) | 16 (23.5) |  | 105 (14.8) | 86 (47.3) | 35 (28.0) |  |
| Gestational age of pregnancy at outcome, mean (SD), wks | 40.0 (1.8) | 39.7 (2.2) | 38.1 (4.1) | <0.001 | 39.8 (2.0) | 39.7 (2.0) | 39.0 (3.2) | 0.001 |
| Preterm birth, n (%) | 48 (5.7) | 22 (12.1) | 14 (21.9) | <0.001 | 53 (7.5) | 12 (6.6) | 24 (19.3) | <0.001 |

BMI= body mass index. ENP= enhanced nutrition package. MUAC= mid-upper arm circumference.

# Table S9. ENAT Cluster-Specific Primary Outcome – Newborn Weight Measured at <72 hrs

| **Health Center** | **N** | **Mean Weight (kg)** | **SD** | **Median Weight (kg)** | **IQR** |
| --- | --- | --- | --- | --- | --- |
| **ENP Health Centers** | 861 | 2.88 | 0.45 | 2.88 | 2.62, 3.13 |
| Korata | 136 | 2.79 | 0.39 | 2.80 | 2.58, 3.03 |
| Arb Geb | 220 | 2.85 | 0.43 | 2.85 | 2.59, 3.12 |
| Ambomeda | 111 | 3.01 | 0.35 | 3.00 | 2.83, 3.16 |
| Forhe | 107 | 2.85 | 0.47 | 2.87 | 2.65, 3.13 |
| Yismala | 146 | 2.91 | 0.59 | 2.93 | 2.56, 3.29 |
| Legidiya | 141 | 2.88 | 0.41 | 2.89 | 2.63, 3.13 |
| **Non-ENP Health Centers** | 749 | 2.90 | 0.44 | 2.92 | 2.64, 3.17 |
| Ambessamie | 149 | 2.91 | 0.38 | 2.90 | 2.66, 3.18 |
| Wanzaye | 26 | 2.95 | 0.61 | 3.04 | 2.67, 3.25 |
| Gelawdios | 84 | 2.90 | 0.44 | 2.95 | 2.64, 3.15 |
| Yifag | 167 | 2.92 | 0.46 | 2.91 | 2.63, 3.20 |
| Kunzla | 130 | 2.85 | 0.41 | 2.90 | 2.63, 3.11 |
| Lalibela | 193 | 2.90 | 0.46 | 2.93 | 2.67, 3.15 |

Live births measured at <72 hours of life, actual measured weights without imputation of missing values.

ENP= enhanced nutrition package. IQR=interquartile range.

# Table S10. ENAT Cluster-Specific Co-Primary Outcome – Newborn Length Measured at <72 hrs

| **Health Center** | **N** | **Mean**  **Length (cm)** | **SD** | **Median Length (cm)** | **IQR** |
| --- | --- | --- | --- | --- | --- |
| **ENP Health Centers** | 839 | 47.8 | 2.9 | 48.3 | 46.4, 49.7 |
| Korata | 132 | 48.0 | 2.5 | 48.3 | 46.9, 49.6 |
| Arb gebaya | 215 | 48.0 | 2.3 | 48.3 | 46.6, 49.6 |
| Ambomeda | 109 | 47.1 | 3.0 | 48.2 | 46.2, 49.3 |
| Forhe | 105 | 47.7 | 2.6 | 48.0 | 46.5, 49.2 |
| Yismal | 141 | 47.7 | 3.5 | 48.2 | 46.1, 50.0 |
| Legidiya | 137 | 48.2 | 3.5 | 49.0 | 46.5, 50.5 |
| **Non-ENP Health Centers** | 709 | 48.3 | 2.8 | 48.5 | 47.1, 50.0 |
| Ambessamie | 144 | 48.7 | 2.2 | 49.1 | 47.8, 50.0 |
| Wanzaye | 26 | 47.1 | 5.0 | 48.2 | 46.0, 49.8 |
| Gelawdios | 83 | 48.3 | 2.9 | 48.6 | 47.0, 50.4 |
| Yifag | 157 | 48.2 | 3.0 | 48.4 | 46.7, 49.9 |
| Kunzla | 121 | 48.8 | 2.4 | 48.5 | 47.4, 50.3 |
| Lalibela | 178 | 47.9 | 2.8 | 48.2 | 46.3, 49.7 |

Live births measured <72 hours of life, actual measured lengths without imputation of missing values.

ENP=enhanced nutrition package. IQR=interquartile range.

# Table S11. Effects of Enhanced Nutrition Package on Pregnancy Outcomes, Including Imputed Missing Outcome Data

|  | **Intervention**  **ENP Arm^a^** | **Control**  **Not-ENP Arm^a^** | **Intervention Effect, unadjusted^b^ (CI^c^)** | **Intervention Effect, adjusted^b,d^ (CI^c^)** |
| --- | --- | --- | --- | --- |
| Total known pregnancy outcomes (n) | 1114 | 1056 | .. | .. |
| Live births (n) | 1089 | 1025 | .. | .. |
| **PRIMARY OUTCOMES** |  |  |  |  |
| Newborn weight^e^, mean (SD), g | 2872 (427) | 2889 (407) | -16.2 (-99.5, 67.1) | -5.7 (-81.7, 70.4) |
| Newborn length^e^, mean (SD), cm | 47.8 (2.7) | 48.1 (2.6) | -0.32 (-0.98, 0.33) | -0.29 (-0.95, 0.37) |
| **SECONDARY OUTCOMES** |  |  |  |  |
| Newborn WAZ^e^, mean (SD) | -0.98 (0.89) | -0.89 (0.87) | -0.10 (-0.28, 0.08) | -0.08 (-0.28, 0.12) |
| Newborn LAZ^e^, mean (SD) | -0.84 (1.3) | -0.61 (1.2) | -0.24 (-0.53, 0.06) | -0.23 (-0.53, 0.08) |

CI= confidence interval. ENP, enhanced nutrition package. LAZ= length for age z-score. WAZ= weight for age z-score.

**^a^** Descriptive statistics are reported at the individual level for each study group.

^b^ Cluster level mean differences are shown for continuous outcomes, and relative risks are shown for dichotomous outcomes.

^c^ For the primary outcomes, 97.5% confidence intervals are reported adjusting for multiplicity (co-primary outcomes) using Bonferroni correction.

^d^ Adjusted for a priori prognostic factors and imbalanced variables: maternal parity, BMI at baseline, height, education, occupation

^e^  n=1089 for the ENP arm and n=1025 for the non-ENP arm.

# Table S12. Effects of Enhanced Infection Management Package on Pregnancy Outcomes, Results Including Imputed Missing Outcome Data

|  | **Intervention**  **EIMP Arm^a^** | **Control**  **Not-EIMP Arm^a^** | **Intervention Effect, unadjusted^b^ (CI^c^)** | **Intervention Effect, adjusted^c,d^ (CI^c^)** |
| --- | --- | --- | --- | --- |
| Total known pregnancy outcomes (n) | 1090 | 1080 | .. | .. |
| Live births (n) | 1070 | 1044 | .. | .. |
| **PRIMARY OUTCOMES** |  |  |  |  |
| Newborn weight^e^, mean (SD), g | 2884 (426) | 2877 (409) | -9.0 (-71.6, 53.8) | -1.39 (-61.2, 58.4) |
| Newborn length^e^, mean (SD), cm | 48.0 (2.6) | 47.9 (2.6) | 0.05 (-0.12, 0.21) | 0.05 (-0.12, 0.22) |
| **SECONDARY OUTCOMES** |  |  |  |  |
| Newborn WAZ^e^, mean (SD) | -0.94 (0.89) | -0.93 (0.88) | -0.03 (-0.17, 0.11) | -0.02 (-0.17, 0.13) |
| Newborn LAZ^e^, mean (SD) | -0.72 (1.2) | -0.74 (1.2) | 0.01 (-0.21, 0.23) | 0.01 (-0.21, 0.24) |

CI= confidence interval. EIMP, enhanced infection management package. LAZ= length for age z-score WAZ= weight for age z-score.

^a^ Descriptive statistics are reported at the individual level for each study group.

^b^ Mean differences are shown for continuous outcomes, and relative risks are shown for dichotomous outcomes.

^c^ For the primary outcomes, 97.5% confidence intervals are reported adjusting for multiplicity (co-primary outcomes) using Bonferroni correction.

^d^ Adjusted for a priori prognostic factors and imbalanced variables: maternal parity, BMI at baseline, height, education, occupation

^e^  n=1070 for the EIMP arm and n=1040 for the non-EIMP arm.

# Table S13. Effects of ENP+EIMP Package on Pregnancy Outcomes, Results Including Imputed Missing Outcome Data

|  | **ENP+EIMP Arm^a^** | **Routine Care (Neither intervention) Arm^a^** | **Intervention Effect, unadjusted^b^ (CI^c^)** | **Intervention Effect, adjusted^b,d^ (CI^c^)** |
| --- | --- | --- | --- | --- |
| Total known pregnancy outcomes (n) | 565 | 531 | .. | .. |
| Live births (n) | 559 | 514 | .. | .. |
| **PRIMARY OUTCOMES** |  |  |  |  |
| Newborn weight^e^, mean (SD), g | 2867 (418) | 2892 (416) | 25.7 (-59.9, 111.3) | 8.15 (-71.2, 87.6) |
| Newborn length^e^, mean (SD), cm | 47.8 (2.7) | 48.1 (2.6) | -.22 (-0.81, 0.37) | -0.2 (-0.76, 0.42) |
| **SECONDARY OUTCOMES** |  |  |  |  |
| Newborn WAZ^e^, mean (SD) | -0.99 (0.89) | -0.89 (0.87) | 0.13 (-0.09, 0.35) | 0.1 (-0.14, 0.34) |
| Newborn LAZ^e^, mean (SD) | -0.86 (1.29) | -0.65 (1.2) | -0.23 (-0.56, 0.1) | -0.21 (-0.55, 0.13) |

CI= confidence interval. ENP= enhanced nutrition package. EIMP= enhanced infection management package. LAZ= length for age z-score. WAZ= weight for age z-score.

**^a^** Descriptive statistics are reported at the individual level for each study group.

^b^ Mean differences are shown for continuous outcomes.

^c^ For the primary outcomes, 97.5% confidence intervals are reported adjusting for multiplicity (co-primary outcomes) using Bonferroni correction.

^d^ Adjusted for a priori prognostic factors and imbalanced variables: maternal parity, BMI at baseline, height, education, occupation

^e^ n= 599 for the ENP+EIMP arm and n=514 for the non-EIMP arm.

# Table S14. Effects of Enhanced Nutrition Package on Birth size: Subgroup analysis MUAC <23 cm

|  | **ENP^a^** | **Not-ENP^a^** | **Unadjusted MD^b^ (CI^c^)** | **Adjusted MD^b,d^ (CI^c^)** |
| --- | --- | --- | --- | --- |
| Total known pregnancy outcomes (n) | 399 | 346 | .. | .. |
| Live births (n) | 398 | 334 | .. | .. |
| Newborn weight^e^, mean (SD), g | 2829 (434) | 2871 (387) | -49.9 (-137, 37) | -39.7 (-164, 85) |
| Newborn weight with imputation, mean (SD), g | 2824 (417) | 2848 (372) | -23.2 (-148.3, 102.0) | -11.6 (-137.5, 114.3) |
| Newborn length^f^, mean (SD), cm | 47.5 (2.9) | 48.3 (2.8) | -0.81 (-1.46, -0.15) | -0.88 (-1.7, -0.1) |
| Newborn length with imputation, mean (SD), g | 47.5 (2.8) | 48.1 (2.5) | -0.65 (-1.26, -0.04) | -0.60 (-1.27, 0.07) |

CI= confidence interval. ENP= enhanced nutrition package. MD= mean difference.

**^a^** Descriptive statistics at the individual level for each study group.

**^b^** Cluster-level analysis

^c^ Primary Outcomes, Confidence Intervals reported are 97.5% given co-primary outcomes with Bonferroni correction; For secondary outcomes, CIs are 95%

^d^ Adjusted for maternal parity, BMI at baseline, height, education, occupation

^e^ Among livebirths, birthweight was measured within <72 hrs for n = 330 (81%) for ENP arm and n = 261 (68%) for non-ENP arm

^f^ Among livebirths, length was measured within <72 hrs for n = 314 (77%) for ENP arm and n = 246 (64%) for non-ENP arm

# Table S15. A priori Sub-group Analysis of Primary Outcomes (ITT)

Analysis with measured <72 hr data, without imputation of missing weight

|  |  | **ENP** | | **not-ENP** | | **ENP vs not-ENP** |  |
| --- | --- | --- | --- | --- | --- | --- | --- |
|  |  | N | Mean (SD) | N | Mean (SD) | aMD (CI) | Interaction p- value |
| **Newborn weight (<72 hrs), g** | | | | | | | |
| Maternal BMI |  |  |  |  |  |  | 0.09 |
|  | <18.5 kg/m^2^ | 168 | 2781 (437) | 102 | 2888 (385) | -136.9 (-333, 59.2) |  |
|  | > 18.5 kg/m^2^ | 692 | 2900 (452) | 643 | 2898 (444) | 16.7 (-44.9, 78.3) |  |
| Maternal age |  |  |  |  |  |  | 0.70 |
|  | <20 yo | 74 | 2798 (400) | 67 | 2789 (390) | -85.7 (-334.6, 163.1) |  |
|  | >20 yo | 787 | 2884 (455) | 682 | 2909 (441) | -7.0 (-81.8, 67.9) |  |
| Primiparity |  |  |  |  |  |  | 0.33 |
|  | Multiparous | 616 | 2913 (459) | 523 | 2942 (442) | -0.02 (-118.8, 83.2) |  |
|  | Nulliparous | 245 | 2784 (416) | 226 | 2800 (412) | 98.7 (-135.4, 332) |  |
| **Newborn Length (<72 hrs), cm** | | | | | | | |
| Maternal BMI |  |  |  |  |  |  | 0.36 |
|  | <18.5 kg/m^2^ | 164 | 47.5 (2.9) | 98 | 48.2 (2.3) | -0.71 (-1.43, 0.01) |  |
|  | > 18.5 kg/m^2^ | 824 | 48.1 (3.1) | 774 | 48.6 (3.1) | -0.23 (-0.95, 0.48) |  |
| Maternal age |  |  |  |  |  |  | 0.45 |
|  | <20 yo | 71 | 47.4 (2.6) | 63 | 47.8 (2.9) | -0.35 (-1.1, 0.5) |  |
|  | >20 yo | 768 | 47.9 (2.9) | 646 | 48.3 (2.8) | -1.0 (-3.6, 1.6) |  |
| Primiparity |  |  |  |  |  |  | 0.27 |
|  | Multiparous | 606 | 47.9 (2.9) | 501 | 48.4 (2.7) | -0.57 (-1.2, 0.08) |  |
|  | Nulliparous | 233 | 47.7 (3.0) | 208 | 47.9 (3.1) | 1.0 (-1.7, 3.7) |  |

aMD= adjusted mean difference. BMI= body mass index. CI= confidence interval. ENP= enhanced nutrition package. ITT= intention to treat.

**Table S16. Maternal Morbidity by Study Arm**

**Supplementary Table 16. Maternal Morbidity by Study Arm**

|  | ENP+EIMP  (n=555) | ENP only  (n=547) | EIMP only  (n=471) | Routine care  (n=498) |
| --- | --- | --- | --- | --- |
| Diagnosed pre-eclampsia, n(%) | 6 (1.1) | 4 (0.7) | 5 (1.1) | 2 (0.4) |
| Diagnosed eclampsia, n(%) | 3 (0.5) | 2 (0.4) | 4 (0.9) | 1 (0.2) |
| Diagnosed Gestational Diabetes, n(%) | 2 (0.4) | 1 (0.2) | 2 (0.4) | 1 (0.2) |
| Diagnosed UTI, n(%) | 16 (2.9) | 24 (4.4) | 16 (3.4) | 22 (4.4) |
| Diagnosed Sexually Transmitted Infection, n(%) | 3 (0.5) | 3 (0.6) | 4 (0.9) | 1 (0.2) |
| Diagnosed Pneumonia, n(%) | 3 (0.6) | 3 (0.6) | 2 (0.4) | 2 (0.4) |
| Diagnosed Tuberculosis, n(%) | 1 (0.2) | 3 (0.6) | 1 (0.2) | 1 (0.2) |
| Diagnosed malaria , n(%) | 7 (1.3) | 5 (0.9) | 2 (0.4) | 3 (0.6) |
| Diagnosed HIV, n(%) | 0 (0.0) | 1 (0.2) | 1 (0.2) | 0 (0.0) |
